# Photoactive Tungsten-Oxide Nanomaterials for Water-Splitting

**DOI:** 10.3390/nano10091871

**Published:** 2020-09-18

**Authors:** Yerkin Shabdan, Aiymkul Markhabayeva, Nurlan Bakranov, Nurxat Nuraje

**Affiliations:** 1 National Laboratory Astana, Nazarbayev University, Nursultan 010000, Kazakhstan; yerkin.shabdan@nu.edu.kz; 2 Faculty of Physics and Technology, AI-Farabi Kazakh National University, Almaty 050040, Kazakhstan; aiko_marx@mail.ru; 3 Faculty of General Education, Kazakh-British Technical University, Almaty 050000, Kazakhstan; 4 Laboratory of Engineering Profile, Satbayev University, Almaty 050000, Kazakhstan; 5 Department of Chemical and Materials Engineering, Nazarbayev University, Nursultan 010000, Kazakhstan

**Keywords:** WO_3_, nanocomposites, heterostructures, water-splitting, oxygen evolution

## Abstract

This review focuses on tungsten oxide (WO_3_) and its nanocomposites as photoactive nanomaterials for photoelectrochemical cell (PEC) applications since it possesses exceptional properties such as photostability, high electron mobility (~12 cm^2^ V^−1^ s^−1^) and a long hole-diffusion length (~150 nm). Although WO_3_ has demonstrated oxygen-evolution capability in PEC, further increase of its PEC efficiency is limited by high recombination rate of photogenerated electron/hole carriers and slow charge transfer at the liquid–solid interface. To further increase the PEC efficiency of the WO_3_ photocatalyst, designing WO_3_ nanocomposites via surface–interface engineering and doping would be a great strategy to enhance the PEC performance via improving charge separation. This review starts with the basic principle of water-splitting and physical chemistry properties of WO_3_, that extends to various strategies to produce binary/ternary nanocomposites for PEC, particulate photocatalysts, Z-schemes and tandem-cell applications. The effect of PEC crystalline structure and nanomorphologies on efficiency are included. For both binary and ternary WO_3_ nanocomposite systems, the PEC performance under different conditions—including synthesis approaches, various electrolytes, morphologies and applied bias—are summarized. At the end of the review, a conclusion and outlook section concluded the WO_3_ photocatalyst-based system with an overview of WO_3_ and their nanocomposites for photocatalytic applications and provided the readers with potential research directions.

## 1. Introduction

The conversion of solar-emitted electromagnetic waves to useful forms of energy is a very promising research area in the field of renewable energy production. Although roughly 32 × 10^24^ J of solar energy reaches the Earth’s surface per year, only 0.001% of the incoming solar energy is used for human needs [1]. The conversion of solar light to useful forms of energy is still challenging at the scientific and engineering level in terms of energy production for the needs of human beings. Even though there are many technologies for renewable energy [2], including solar cells, solar collectors and solar fuel reactors (water-splitting), the major challenges we face are to improve efficiency and stability in the conversion of solar energy to other energy forms. Currently, one of the popular research technologies to tackle solar energy conversion is trying to convert photons into chemical energy [3] by using artificial photoelectrochemical (PEC) processes.

Metal-oxide nanomaterials have been thoroughly studied for the conversion of solar energy to hydrogen molecules due to their chemical and physical stability, optical and electronic properties, easy fabrication and low cost. They have shown good properties for use in photoelectrochemical devices such as TiO_2_ [4,5,6], α-Fe_2_O_3_ [7,8,9], BiVO_4_ [10,11,12], ZnO [13,14,15] and WO_3_ [16,17,18]. On the above properties of semiconductor materials, both suitable bandgap positions to generate hydrogen and oxygen gases and the ability to absorb a reasonable portion of the solar light spectrum are critical for water-splitting. However, a single metal-oxide photocatalyst cannot simultaneously satisfy all the requirements for solar-to-hydrogen-driven systems since it encounters many problems (including fast recombination of charge carriers, photo corrosion, instability in aggressive electrolytes, short lifetime of charge carriers, improper bandgap or diffusion length of photogenerated electrons and holes). As a result, most of the metal oxide semiconductors are not suitable to split water at the visible light irradiation, which occupies 54% of whole solar spectrum since they either do not have proper bandgaps or only absorb the UV light region. However, the problems stated above have been successfully addressed by introducing heterojunctions, composite nanomaterials, coupling wide band and narrow band materials, doping, surface–interface engineering, dye sensitization, etc.

Among the metal oxides, WO_3_ is a promising semiconductor for PEC water-splitting with favorable properties. (These properties include: suitable bandgap (~2.6 eV), good chemical stability under strong solar exposure, oxygen-evolution capability, long minority carrier diffusion length (~500 nm–6 μm [19,20]), absorption of visible light (~12%) and low cost.) The conduction band energy position of WO_3_ is 0.25 eV, which is not suitable for reorientation of bonds of hydrogen atoms from the aqueous phase to the gaseous (0 V vs. NHE). On the other hand, the valence band, located at 2.7 eV, is more positive than the oxidation potential of oxygen (1.23 V vs. NHE) and is suitable for oxygen evolution. Although the WO_3_ photocatalyst suffers from some limitations such as sluggish charge transfer [21], boosting charge separation can be achieved by modifying WO_3_ photoanode with numerous materials including Ag nanoparticles [22] and Au plasmonic particles [23]. Many papers have reported on using WO_3_ photoanodes for O_2_ evolution study [24,25]. During the study of hydrogen evolution from aqueous phase, various photoelectrochemical systems and configurations integrated with WO_3_ and its composites have been developed.

Among the published materials in this prospect, numerous amounts of work can be distinguished: Ji et al. reported a triple layer heterojunction BiVO_4_/WO_3_/SnO_2_ material with a perovskite solar cell [26], Liu. et al. prepared a WO_3_ photoanode with a tandem cell [27] and Lee used dye-sensitized solar cells to produce hydrogen with bare WO_3_ photoanodes [28]. Zhang fabricated the WO_3_@a–Fe_2_O_3_/FeOOH photoanode, which exhibits a 120 mV negative shift in onset potential and yields a photocurrent density of 1.12 mA/cm^2^ at 1.23 V vs. reversible hydrogen electrode (RHE) [29]. Moreover, some systems use free-particle WO_3_ heterostructures based on photochemical cell reactions. Despite the fact that WO_3_ cannot generate hydrogen, there are some publications where scientists show high photocatalytic activity for CdS-WO_3_ [30] and non-stoichiometric WO_3−x_/CdS heterostructures for efficient hydrogen generation [31].

**Figure 1:**
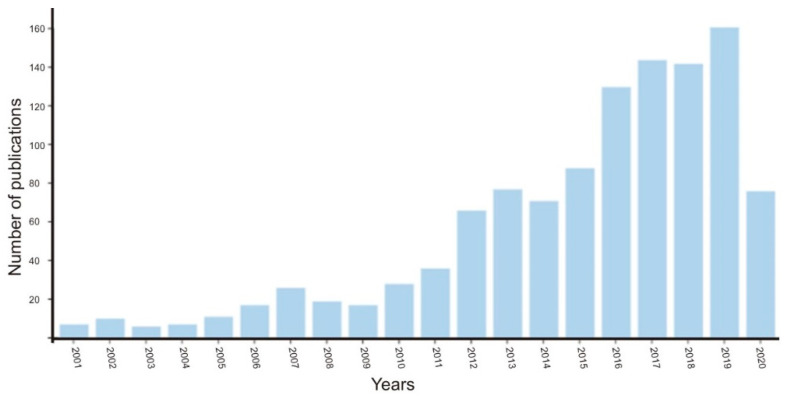
Statistics analysis from Web of Science indicates increase of recent publications in WO_3_ photocatalytic areas.

Thus, we have conducted a literature survey on the WO_3_-based photocatalytic system and found a dramatic increase of publications recently (Figure 1). This indicates that the WO_3_ is a very important material for designing efficient photocatalytic systems. The analysis of scientific articles, reviews and conference materials found in the authoritative database revealed few review papers in the use of tungsten trioxide photocatalyst for water-splitting. As shown in Figure 1, the trend of the published papers in the WO_3_ photocatalytic research is increasing exponentially. Therefore, in our opinion, it is essential to present a review article to our scientific community with recent research progress of WO_3_ in the photocatalytic water-splitting. Although there are some review papers that included the WO_3_ and their water-splitting applications, from the best of our knowledge, few papers have been specifically focused on sole WO_3_/nanocomposites and their recent photocatalytic application.

The primary focus of our review article is to deliver the recent progress of tungsten-based photocatalytic systems that have been developed. More specifically, it discusses the morphology, crystal, doping, surface–interface engineering effect of WO_3_ on the heterostructured photocatalytic system, and all of the results in different conditions including electrolyte, power, applied bias, morphology, and synthesis approaches were tabulated for the researchers to check. Therefore, in this review, we try to give comprehensive information on WO_3_ including the physical chemistry property, crystal structure, and nanomorphology along with their composites including binary and ternary structures used in the particulate, PEC, Z-scheme and tandem configuration for effective water-splitting applications.

## 2. Basic Principles of the Water-Splitting Reaction

### 2.1. Thermodynamics of Water-Splitting

In the reaction of water-splitting, solar energy can be directly converted into chemical energy form, hydrogen gas [32,33,34]. The hydrogen acts as a green energy carrier since it possesses high energy density. When used in a fuel cell, water is the only byproduct.

As early as 1923, J. B. S. Haldane, a British scientist, proposed a concept of photocatalytic hydrogen production. Seeing that there is no naturally produced pure hydrogen on Earth, its resource is highly abundant throughout the universe. Like fossil fuels, water or biomass can be utilized to produce hydrogen or other chemical fuels. Hydrogen gas can be further used in hydrogen fuel cell-powered vehicles, which are much more environmentally friendly than the commonly used nonrenewable fuel options. Increasing the efficiency of water-splitting devices for hydrogen fuel production has a potential to decrease its dependence on using fossil fuels and importation.

There are some other approaches for hydrogen production, however, the most environmentally sustainable, “green” method is photocatalytic or photoelectrochemical water-splitting. The PEC water-splitting works similarly to a solar cell. The main difference is that it converts solar energy to a chemical bond instead of converting directly to electric power, which is beneficial to store energy for later use. PEC consists of three main components: an anode, a cathode and an electrolyte (aqueous media). At the anode, water is oxidized to generate oxygen via the oxygen evolution reaction (OER), whereas at the cathode hydrogen ions are reduced into hydrogen gas via a hydrogen evolution reaction (HER). Based on the configuration of the PEC cell, either the cathode or anode, or both, can be photoactive semiconductors which absorb light. Furthermore, water can also split via connecting a p–n junction solar cell in parallel with a photoelectrochemical cell. This process not only avoids the complicated manufacturing process, but it also reduces the system’s cost [35]. Although extensive research has been conducted using many semiconductor configurations, there is still so much that needs to be done to reach the targeted efficiency and stability goals. For a particle-based photo catalytical system, the ideal solar to hydrogen (STH) efficiency should be 10% [36,37]. This efficiency brings cheaper H_2_ production.

In 1972, Japanese scientists, Fujishima and Honda first studied TiO_2_ as a photonic material and proved that water can be decomposed under UV-light exposure [38]. Since then, scientists have been studying a variety of light-sensitive material, including all inorganic and organic dyes [39,40,41].

Decomposing water into H_2_ and O_2_ is an endothermic reaction thermodynamically (+237.2 kJ/mol). This means that additional energy is required to perform the decomposition reaction (E-1):

ΔG^0^ = −nFΔE^0^ = +237.2 kJ/mol H_2_ (1)

where:

F—Faraday’s constant (F = 96,485 C/mol),

n—Number of transferred electrons (n = 2)

ΔE^0^—standard potential of the electrochemical cell (ΔE^0^ = 1.229 V).

The amount of Gibbs free energy required to split a molecule of water into hydrogen and oxygen is ΔG = 237.2 kJ/mol, which is corresponded to ΔE^0^ ≈ 1.23 eV per electron, transforming the Nernst equation under standard conditions. This means a minimum energy of 1.23 eV per electron should be supplied by the photocatalyst. This process can be written in the following two half-reactions (E-2; E-3; E-4):

Water oxidation: H_2_O + 2h^+^ → ½ O_2_ + 2H^+^ (HER) (2)

Water reduction: 2H^+^ + 2e^−^ → H_2_ (ORE) (3)

H_2_O → ½ O_2_ + H_2_ ΔG = +237.2 kJ/mol (4)

The bandgap (Eg) is the main parameter that defines the light-harvesting ability of an absorber. Photons alone with energies higher than the bandgap can excite electrons in the valence band to the conduction band. The excess energy or the difference in the energy of the absorbed photon and the band gap energy (𝐸–𝐸_𝑔_), is lost as phonons. The absorption coefficient of the semiconducting materials is another parameter which shows how efficiently a photocatalyst can harness the solar spectrum. One crucial point that needs to be taken into consideration as quantifying the optimal minimum band gap value is the intrinsic loss (E_loss_), associated with the solar energy conversion process. These losses are connected with the fundamental loss caused by thermodynamics because of non-ideality (kinetic losses) in the conversion process [35,38]. The former loss results from the second law of thermodynamics. In fact, the following equation shows how bandgap energy (Eg) corresponds to the change in internal energy, which is related to the change in Gibbs energy (E-5):

ΔG = ΔU + PΔV − TΔS(5)

where U, P, V, T and S indicate the internal energy, pressure, volume, temperature and entropy, respectively. When the semiconductor absorbs photons, increasing excited states can be created in addition to ground states, increasing the entropy of the ensemble. The change in entropy or ΔS_mix_, occurs because there are existing excited states along with the ground states. A volume change (ΔV_mix_) is also caused by the mixture of excited and ground states. However, this is not true for the ideal chemical system (ΔV_mix_ = 0). Thus, the band gap energy should be greater than the available work under ideal conditions (Gibbs energy change per electron), at least E_loss_ = TΔS_mix_ with a minimum of 0.3–0.5 eV. In reality, E_loss_ reaches higher values (roughly 0.8 eV) as a result of kinetic losses and due to non-ideality (overpotential at the anode and cathode, reduction in resistance at the electrolyte, electron–hole pair recombination). Therefore, in order to maximize the chemical conversion efficiency, materials commonly used as photoelectrodes in PEC cells require a band gap of 2.0 to 2.25 eV [35,38].

**Figure 2:**
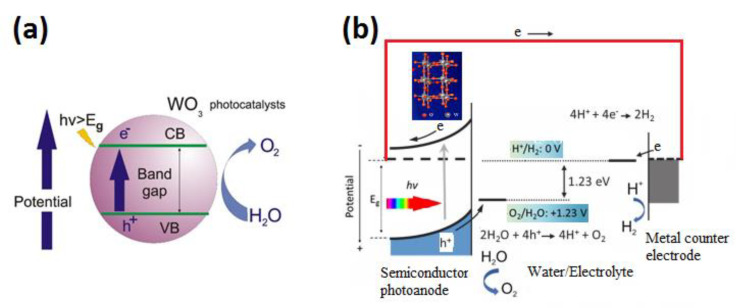
(**a**) Schematic illustration for WO_3_-particle-based photocatalyst system; (**b**) principle of photoelectrochemical water-splitting.

When UV and/or visible sunlight shine onto a semiconductor photocatalyst, the semiconductor absorbs photons and excites electrons from the semiconductor’s valence band to its conduction band, leaving a hole in the valence band, i.e., electron–hole pairs (Figure 2a). This is the so-called, “photo-excited” semiconductor phase. The bandgap is the difference between the maximum valence band energy and the minimum conduction band energy. Ideally, semiconductors have a bandwidth greater than 1.23 V as well as a more negative conduction band relative to the water reduction potential and a more positive valence band relative to the water oxidation potential.

A typical one-step PEC configuration for water decomposition consists of either a photoanode or a photocathode. N-type tungsten trioxide is mostly represented as a photoanode and the basic principles of such cell can be depicted in Figure 2b. Process of PEC water decomposition is initiated via accepting light photons by photoactive materials. Then, this step is accompanied by generating excitons (electron–hole pairs) inside semiconductors. Photogenerated holes on the surface of WO_3_ can oxidize water while electrons flow to the Pt electrode to produce hydrogen (Figure 2b). Due to the improper positioning of the conduction and valence bands with respect to the potentials of water reduction and oxidation, external bias voltage is used to separate excitons.

Another thermodynamic precondition is the position of the band edges. For the oxidation reaction to occur, holes move from the photoelectrode to the interface between the semiconductor and the solution freely. The top edge of the valence band must be more positive than the oxidation potential of O_2_/H_2_O as seen in Figure 2b. Likewise, the reduction reaction happens if the bottom edge of the conduction band is more negative than the reduction potential of H^+^/H_2_.

**Figure 3:**
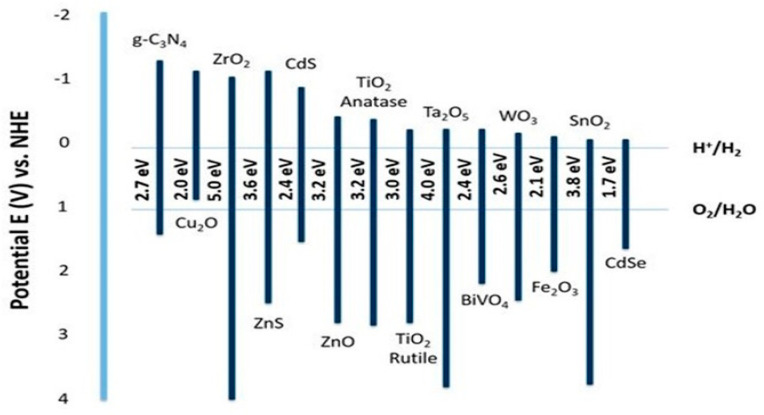
Bandgap positions of typical semiconductors for water-splitting. Reproduced from [42], with permission from MDPI, 2016.

Figure 3 shows the band structure and bandgap values of some semiconductors [42]. While wide band gap (Eg > 3 eV) photocatalyst can harvest only UV light (a small portion of the solar spectrum, less than 4%), its band gap can be easily engineered to absorb the visible light range via metal and nonmetal doping. Furthermore, narrow bandgap materials (e.g., WO_3_, Fe_2_O_3_) are not able to drive the water reduction and oxidation reactions at the same time since their bandgap energy positions are not properly positioned to the water redox potentials. Therefore, they are commonly used to construct tandem cell structures for the water-splitting reaction.

### 2.2. Device Requirements and Calculation of Their Efficiency

Various types of overall water-splitting techniques include: particulate systems [43], Z-schemes [44,45,46] and photoelectrochemical cells [46]. The photoelectrodes of PEC water decomposition such as a photoanode and a cathode electrode are made up of photocatalysts. However, fabrication of stable photoelectrodes under the influence of strong sunlight is still a challenging one in PECs. Right now, it is preferred to fabricate the film via direct growth on the photoelectrode, which provides a relatively stable photoactive films.

In order to design an efficient and affordable solar hydrogen production PEC system, the electrode requires low cost materials with the capability of efficient light harnessing and long term stability.

To date, large band gap semiconductors (UV-active specifically) and metal oxides have been extensively investigated for the photocatalytic water-splitting studies due to their robustness and suitable band gap energy positions. One challenge to using these materials is the limitation of solar light harnessing to a small portion of the solar spectrum.

The following formula helps us calculate the theoretical maximum photocurrent J_max_, which is proportional to the solar–hydrogen conversion efficiency (STH):

J_max_ = q ∫ Φ_λ_ [1 − exp(−α_λ_d)] dλ (6)

where λ, q, d and α_λ_ represent wavelength, electron charge, sample thickness and absorption coefficient under the photon flux of the AM 1.5 G solar spectrum, respectively. Considering the conversion, reflection and other losses, obtaining the goal of 10% STH conversion efficiency is very challenging.

The following efficiencies are usually reported for PEC cells. They are STH conversion efficiency, applied bias photon-current efficiency (ABPE), external quantum efficiency and internal quantum efficiency (IQE) or absorbed photons to current efficiency (APCE).

STH efficiency is commonly used to evaluate PEC device performance and is expressed in the following way:

STH = [(H_2_ production rate) × (Gibbs free energy per H_2_)]/([incident energy]) (7)

The E-8 formula can be applied to calculate ABPE:

ABPE = [J_ph_ × (1.23 − V_b_)]/P_total_ (8)

where J_ph_ is the photocurrent density as a bias V_b_ is applied and P_total_ is the total incident solar light power.

External quantum efficiency defines the photocurrent generation per incident photon flux under a certain irradiation wavelength. Solar-to-hydrogen conversion efficiency can be evaluated via applying the external quantum efficiency data over the total solar spectrum in a two-electrode system. However, applying external quantum efficiency data obtained in a three-electrode system under a bias to estimate solar-to-hydrogen conversion efficiency is not considered to be a valid method. However, it is still considered to be a useful approach for finding PEC cell material properties. The external quantum efficiency (EQE) is expressed by equation (E-9):

EQE = (J_ph_ × hc)/(P_mono_ × λ) (9)

where J_ph_ is the photocurrent density, h is Planck’s constant, c is the light speed, P_mono_ is the power of calibration and monochromatic illumination, and λ is the wavelength of monochromatic light.

## 3. WO_3_ and Its Nanocomposites for Particle-Based Photocatalytic Systems

### 3.1. Half Reaction Systems

Nowadays, the pursuit for highly efficient photocatalytic materials to produce hydrogen fuel under the exposure of light photons is still in the active stage. In a photoelectrochemical cell, it is required to create sufficient voltage between the anode and cathode to perform the water decomposition reaction. However, most of wide bandgap semiconductor materials are not able to respond to the visible part of the spectrum. Absorption of ultraviolet radiation alone is an undesirable property of photocatalyst operating in terrestrial conditions. One of the exciting ways to solve the above contradictions is the creation of photocatalytic systems consisting of a series of photocatalysts. That is why researchers try to use photochemical systems, where water can be decomposed using colloid particles without any external voltage. Many papers have reported [47] that hydrogen can be generated, even though the efficiency is very low. Evolution of oxygen is difficult because it requires process of four electrons and four H^+^ transfers.

Under solar illumination, although photoexcited electrons and holes are produced, they simultaneously experience recombination and back reaction, which are competitive processes of photogeneration. Hence, most works focus only on half reactions where either H_2_ or O_2_ evolution is possible in the presence of sacrificial electron donor or acceptor.

C_d_S/WO_3_ photocatalysts produced a high hydrogen evolution rate of 369 μmol/gh with lactic acid as an electron donor [30]. Further modification of C_d_S/WO_3_ with Pd particles increased the hydrogen evolution rate to 2900 μmol/gh, 7.9-fold higher than for C_d_S/WO_3_.

**Figure 4:**
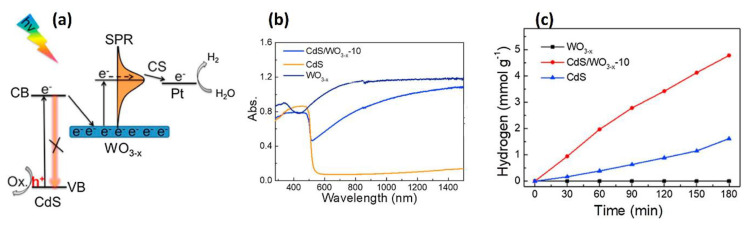
(**a**) Graphic illustration of charge transfer for CdS/WO_3−x_ composite; (**b**) DRS spectra of WO_3−x_, CdS and CdS/WO_3−x−10_; (**c**) hydrogen generation for CdS nanowires, WO_3−x_ and CdS/WO_3−x−10_ composites in 20 vol% lactic solution under illumination. Reproduced from [31], with permission from Elsevier, 2018.

Furthermore, the surface plasmon resonance (SPR) effect of non-stochiometric WO_3−x_ was demonstrated [31] from C_d_S/WO_3−x_ heterostructures photocatalysts via photoinduced electron injection for hydrogen evolution. The non-elemental metal plasmonic material WO_3−x_ has intense SPR in the visible/NIR region (Figure 4b). Free electrons in the conduction band of WO_3−x_ can be generated from oxygen vacancies that are results of chemical reduction during synthesis. Further excitation of electrons can happen by SPR and then they can become hot electrons for the hydrogen generation as shown in Figure 4a. Photo-excited electrons on CdS inject into conduction band of WO_3−x_, so that the SPR of the photocatalyst WO_3−x_ is stable and some hot electrons participate in hydrogen evolution reactions (Figure 4c).

In addition, to choose the photoanode material for the half-reaction of water-splitting, attention should be paid to the selection of the electrolyte. For sulfide semiconductors and composites, a Na_2_S/Na_2_SO_3_ mixture is used as an absorbing hole agent. In type II heterojunctions, for example, WO_3_–NS/C_d_S–NR, with high conductivity, WO_3_ provides efficient charge collection and, therefore, reduces the rate of space charge recombination, which leads to the accumulation of holes in cadmium sulfide. An electrolyte based on Na_2_S/Na_2_SO_3_ provides fast hole collection, which allows the half-reaction to occur without degradation of the photoanode [48]. The effect of some electrolyte solutions on the oxidative half-reactions of WO_3_-based photoanodes was studied on [49]. James C. Hill and Kyoung-Shin Choi studied photo-oxidative processes in chloride solutions, acetate solutions, phosphate solutions, perchlorate solutions, sulfate solutions and solutions with K^+^ and Li^+^ cations. The electrodeposited porous WO_3_ layers were used as a photoanode. The results show that the presence of acetate and chloride ions suppressed the release of O_2_. In a phosphate solution, the release of O_2_ and the formation of peroxides was the main result of photooxidation. The oxidation of water in perchlorate electrolytes was accompanied by the release of O_2_ and the formation of peroxides. In this case, the photocurrent density in such a system was lower in comparison with phosphate electrolytes. The authors also showed that cations have a significant effect on the efficiency of conversion of the photocurrent to O_2_. For example, Li^+^ ions adsorbed on the surface of WO_3_ serve as blockers of water oxidation centers, while K^+^ ions increase oxygen evolution in perchlorate, sulfate and phosphate solutions.

The effect of tungsten trioxide layers on hydrogen reduction processes also demonstrates positive dynamics. When combining Cu_2_O with WO_3_, a semiconductor p–n junction is created and that generates the conditions for the rupture of photogenerated excitons. Thus, in the Cu_2_O/WO_3_ heterostructure, an enhancement of the half-reaction of reduction is demonstrated in comparison with the sole Cu_2_O photocatalyst.

### 3.2. Z-Schemes

**Figure 5:**
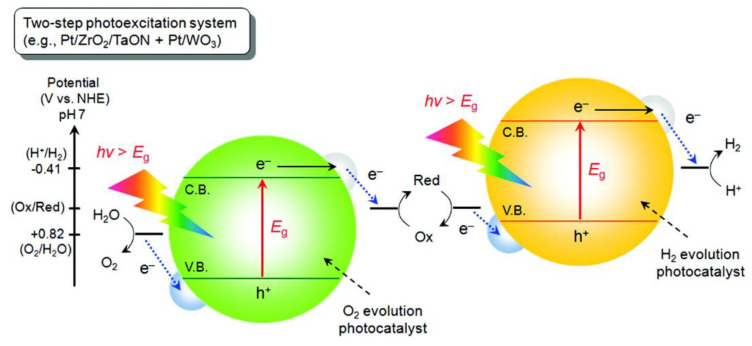
Schematic illustration of photocatalytic water-splitting by Z-system. C.B.—conduction band; V.B.—valence band; Eg—bandgap. Reproduced from [50], with permission from American Chemical Society, 2010.

Z-scheme photocatalysts for overall water-splitting are a combined system involving two photon excitation processes (Figure 5) [50]. The two–photon excitation system was proposed by Bard et al. in 1979 which mimicked natural photosynthesis [51]. A Z-scheme is composed of one H_2_ evolution photocatalyst and another O_2_ evolution photocatalyst with electron mediator. Most of Z-scheme construction was demonstrated using Pt co-catalyst loaded SrTiO_3_, TaON, CaTa_2_O_2_ N and BaTa_2_O_2_N for hydrogen evolution and Pt/WO_3_ for oxygen evolution. Photocatalytic activity depends on pH level, concentration of electron mediator and type of co-catalysts. For example, Hideki Kato showed that pH level affects photocatalytic activity of Z-scheme consisting of Pt/SrTiO_3_Rh–WO_3_–FeCl_3_ system [52]. The pH value was adjusted using sulfuric and perchloric acids between 1.3 and 2.5. It was shown that the best photocatalytic activity was achieved at pH 2.4 and subsequent increasing of pH led to decrease of the activity. The sulfate ions-induced formation of [Fe(H_2_O)_5_(SO_4_)]^+^ species around pH 2.4. Under 48 h of solar illumination, the Z-scheme generated both 890 and 450 μmol H_2_ and O_2_, respectively.

Yugo Miseki reported a Z-scheme system with an oxygen evolution photocatalyst of PtO_x_/H–Cs–WO_3_ [53]. The Z-scheme water-splitting efficiency with PtOx/H–Cs–WO_3_ was 3-fold higher than that of using PtO_x_/WO_3_. Adding Cs^+^ ions to the PtO_x_/WO_3_ significantly improved the oxygen evolution rate. IO_3_^−^ ion was used as an electron acceptor in this work. The apparent quantum yield at 420 nm was 20% which is the best index among photocatalysts using the IO_3_^−^ redox.

Another Z-scheme, containing g-C_3_N_4_–WO_3_ photocatalysts, demonstrated enhanced H_2_ evolution [54]. The high photocatalytic activity is most likely due to direct electron transfer from WO_3_ to g-C_3_N_4_ in the Z-scheme.

One Z-scheme system consisting of graphitic nitrite g-C_3_N_4_ and WO_3_ nanocomposites modified with co-catalyst Ni(OH)_x_ showed the highest hydrogen production rate of 576 μmol/(g.h). Photogenerated electrons and holes are efficiently separated by combination of g-C_3_N_4_ and Ni(OH)_x_. The electron spin resonance (ESR) technique used DMPO (5,5-dimethyl-1-pyrroline N-oxide) as a trapping agent of •O^2−^ and •OH radicals to register the change of the active oxidizing species in aqueous systems. As a result, the Z-scheme charge separation mechanism explained the high hydrogen production rate [55].

Recently, construction of Z-scheme using ZnO–WO_3−x_ nanorods was successfully synthesized by the solvothermal method [56]. A high photocurrent value of 3.38 mA/cm^2^ at 1.23 V vs. RHE, which is 3.02-fold higher than pure ZnO, was obtained by an effective Z-scheme charges-transfer process. Red shift of optical absorption and better electrochemical performances were achieved by decoration of ZnO nanorods with WO_3−x_ nanoparticles.

Sayama et al. reported [57] a stoichiometric production of H_2_ and O_2_ using a mixture of Pt-WO_3_ and PtSrTiO_3_ (Cr–Ta-doped) in NaI media. The Pt-loaded SrTiO_3_ (Cr–Ta-doped) produced H_2_ of 0.8 μmol h^−1^ from an aqueous NaI solution while the Pt-loaded WO_3_ produced O_2_ at an initial rate of 84 μmol h^−1^ in an aqueous NaIO_3_ solution under visible light (l > 420 nm) separately. The H_2_ evolution rate from the mixed photocatalyst system (1.8 μmol h^−1^) was higher than that from Pt–SrTiO_3_ (Cr–Ta-doped) alone (0.8 μmol h^−1^), indicating that addition of the Pt–WO_3_ effectively reduced the IO^3−^ ion to I^−^.

Even though the band position of WO_3_ is suitable for O_2_ evolution, doping WO_3_ with a metal can shift the energy level. Wang [58] studied electronic properties of WO_3_ using density functional theory (DFT) calculations with a hybrid calculation. Replacing W by Mo and Cr in the lattice can modify the bandgap of WO_3_ and improve absorption of visible light. The effect of replacing O atoms by S anions was simulated by substitution along the Z direction in the unit cell. The DFT results predicted that there is a decrease in energy gap (2.21 eV) as well as a conduction band shift up, which is beneficial for HER. The authors also tested the effect of doping WO_3_ with Ti, Zr and Gf metals, resulting in a predicted upward shift of the conduction band like the case with S anions.

Mg-doped WO_3_ photocatalysts [59] have been studied experimentally. The conduction band edge for p-type Mg-doped WO_3_ was −2.7 eV vs. Saturated calomel electrode (SCE) at pH 12, which is more negative than the reduction potential of H_2_. Hydrogen generation of 3 μmol/gh was achieved by doping WO_3_ with Mg (5–10 wt%). Doping has also been done using other metals, including Mo [59].

## 4. Heterostructured WO_3_ Nanocomposites for Photoelectrochemical Cell Systems

Photocatalytic activity of WO_3_ depends on the crystal structure, morphology and surface areas. High surface areas of WO_3_ usually increase the photo activity via providing more reaction sites. The certain morphology increases electron mobility, thus demonstrates better photocatalytic activity. For example, one dimensional WO_3_ demonstrates relatively high photoactivity relative to nanoparticles. In the two dimensional WO_3_ nanomaterials, it is very important to have optimum grain size which lead to high photoactivity. The crystal structure is critical for the photoactivity of WO_3_. Furthermore, monoclinic structure of WO_3_ offers different photocatalytic activity relative to other crystalline structure including tetragonal, etc.

Anodization [60,61], solvothermal [56], hydrothermal [62,63], spin coating [64], electrodeposition [65,66] and sol–gel [67] methods were used to fabricate different morphologies and structures.

### 4.1. Crystalline Structure

**Figure 6:**
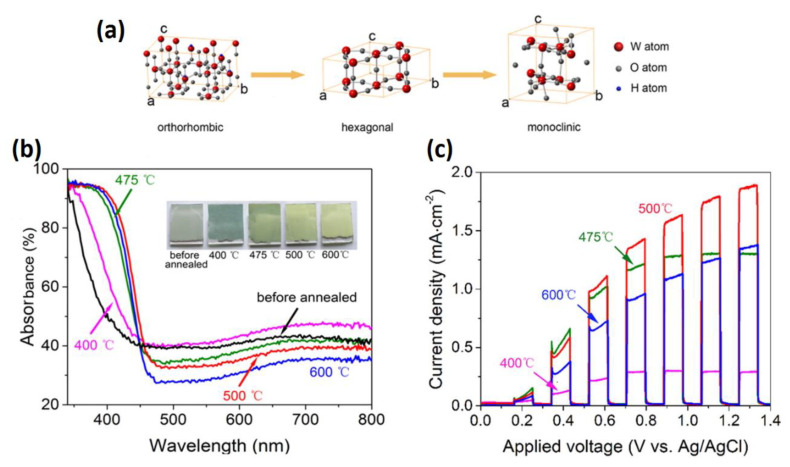
(**a**) Crystal-unit cells for orthorhombic WO_3_·0.33H_2_O, hexagonal WO_3_ and monoclinic WO_3_; (**b**) absorption peaks of WO_3_ films with/without heat treatment; (**c**) linear-sweep voltammetry of WO_3_ photoanodes at different temperatures under chopped illumination. Reproduced from [68], with permission from American Chemical Society, 2016.

Many research efforts have been performed to investigate the effect of crystal structure on the tungsten photocatalytic activity. It was found that the monoclinic crystalline phase demonstrated stronger oxidation activity than other crystal phases such as hexagonal and orthorhombic. The monoclinic phase was found to be the most stable at room temperature [68,69,70,71,72]. Increase of the temperature gradually transformed WO_3_·0.33H_2_O from orthorhombic into anhydrous hexagonal and a final stable form monoclinic (Figure 6a). As the temperature transited from 400 to 500 °C, the color of the film turned into yellowish color obviously, which is corresponded to a red shift (Figure 6b). The photocurrent density increases until 500 °C, then it starts to decrease (Figure 6c). The monoclinic structure of WO_3_ at 500 °C showed the highest photoelectrochemical performance, on the contrary the orthorhombic WO_3_·0.33H_2_O exhibited the lowest photocurrent density [68,69,70,73,74,75].

**Figure 7:**
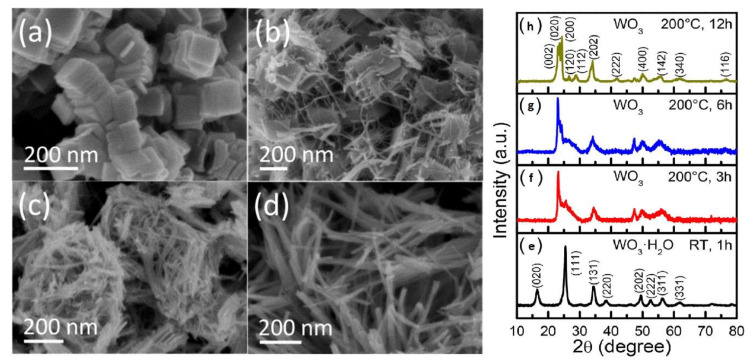
SEM images. (**a**) Stacked WO_3_·H_2_O square nanoplates synthesized at room temperature, WO_3_ nanowires evolving after (**b**) 3 h, (**c**) 6 h and (**d**) 12 h solvothermal treatment of WO_3_·H_2_O nanoplates at 200 °C; (**e**–**h**) corresponding XRD patterns. Reproduced from [76], with permission from American Chemical Society, 2017.

Nayak et al. [76] used combination of a facile precipitation and solvothermal methods to fabricate WO_3_ nanowires. The precipitation method produced (Figure 7a–d) WO_3_·H_2_O nanoplates with an orthorhombic phase, later the solvothermal approach was used to form WO_3_ nanowires with a monoclinic phase (Figure 7e–h). The photocurrent density obtained from WO_3_ monoclinic structure was 21-fold higher than that of WO_3_·H_2_O orthorhombic phase. This enhancement was ascribed to the presence of different phases between WO_3_·H_2_O nanoplates and WO_3_ nanowires or the high crystallinity of WO_3_ nanowires, which minimized the barrier of charge transfer at the interfacial charge and enhanced the PEC performance.

The effects of crystal phase on the photocatalytic performance has been broadly explored. Park et al. [77] found that the annealing treatment reduced the surface disorder induced by water via improvement of the crystallinity or oxygen deficiencies of WO_3_, led to enhancement of the PEC performance. Zeng’s group explained the formation of peroxo species on the surface of WO_3_·H_2_O as it has low degree of crystallinity. As the annealing temperature of WO_3_ reached 500 °C, highly reactive (002) facets were formed to reduce defects, thus to minimize the recombination of electron–hole pairs [75]. The same conclusion was obtained by Su’s group [72]. From the above studies, monoclinic WO_3_ demonstrated higher PEC performance than that of as-prepared hydrated WO_3_.

Recent investigations demonstrated that with surface engineering certain crystal planes possess preferences on the photoexcited electrons and holes, which lead to either preferential oxidation or preferential reduction reactions [78]. Furthermore, photo–electrochemical efficiency has been improved via exposing the high surface crystalline surface [79,80]. Among the three crystal planes or facets of WO_3_ which are (200) with 1.43 J/m^2^, (020) with 1.54 J/m^2^ and (002) with (1.56 J/m^2^) facet of WO_3_, the crystal facet (002) showed preference for adsorbing the reaction species due to its highest surface energy [81]. Wang et al. confirmed this via DFT calculations [82]. The dangling O atoms of the weakest W–O bond on the (002) crystal plane of the monoclinic WO_3_, offer plentiful active sites for H_2_O and organic molecules through the hydrogen bond. Oxidization of water and degradation of organics on the (002) easily occur via consuming photo-excited holes and generating active oxygen species, which reduce the recombination of photogenerated carriers [83,84,85].

The morphology of the WO_3_ films can be controlled by synthesis parameters such as synthesis time, temperature and the amount of the capping agent [75]. HRTEM study revealed that annealing WO_3_·H_2_O plates transformed along with the (020) crystal face into WO_3_ plates with preferentially (002) facet. At annealing 500 °C, the WO_3_ showed 1.42 mA cm^−2^ at 1.23 V vs. RHE, which is relatively high current density. This is explained due to reduction of peroxo species on the surface of WO_3_. The high energy crystal plane of WO_3_ nanoplate enhanced PEC water-splitting. Zhang et al. [83] compared monoclinic WO_3_ nanomultilayers which has preferable (002) facet with that of WO_3_ nanorods and found that WO_3_ nanomultilayers performed higher photocurrent densities than the WO_3_ nanorods. These results were explained not by the specific surface area of WO_3_ nanorods, but the presence of highly reactive (002) facets of WO_3_, which contributed to the improved PEC water-splitting performance.

In addition, increasing studies have been made investigating the effect of the (002) crystal plane of 2D monoclinic WO_3_ on PEC water-splitting.

To enhance the PEC water-splitting performance of WO_3_, most of studies have been focused on engineering morphology, crystallinity, heterojunction, oxygen vacancy, doping and co-catalysts for enhancement of photocatalytic hydrogen evolution.

According to the crystalline structure of WO_3_, it is confirmed that the monoclinic phase of WO_3_ demonstrated higher OER than the hexagonal or orthorhombic phases since it is the most stable phase at room temperature and presence of highly reactive (002) facets.

### 4.2. Morphologic Effect

Various WO_3_ nanomaterials with different morphologies including nanorods [86], nanoflake [87], nanotubes [88,89], nanoplates [90] and nanoparticles [91,92] were synthesized by various methods to provide active sites for catalysis. It was found that morphology change of WO_3_ can significantly influence photocatalytic activity.

Ma and other authors [93] obtained nanoplates of WO_3_ by topological method using Na_2_WO_4_ and HBF_4_ and mentioned that intrinsic crystal lattice of tungsten acid plays important role to obtain morphology of final products. The crystal lattice of H_2_WO_4_ has (WO_6_) octahedra layers with normal direction (010) and each layer is linked to each other via hydrogen bonds. That is why H_2_WO_4_ tends to form platelike nanocrystals with (010) direction. Another factor affecting morphology is addition of directing agents for nucleation and crystal growth. Interaction of H_2_WO_4_ crystal planes and HBF_4_ can be reason of formation plate morphology of WO_3_. Meng and others [94] synthesized hierarchical structure using citric acid C_6_H_8_O_7_ and found that (–COOH) functional groups affect growth of nanoplate. They concluded [93,94] that uniform platelike morphology is favorable for gas sensing because it has more active sides for absorption of gas molecules. In addition, much work was done on WO_3_ crystal growth using fluoroboric acid [95], polyethylene glycol (PEG) [96], polyvinyl alcohol (PVA) [87].

**Figure 8:**
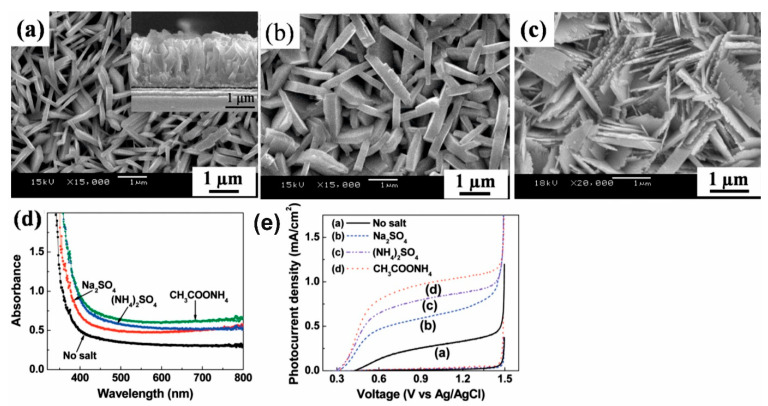
SEM images for WO_3_ hydrate film with sheetlike nanostructures with (**a**) (NH_4_)_2_SO_4_, (**b**) Na_2_SO_4_ and (**c**) CH_3_COONH_4_ as capping agent; (**d**) absorption spectra of WO_3_ films; (**e**) current-potential scans of WO_3_ films measured in darkness and under illumination in a 1-M H_2_SO_4_ electrolyte containing 0.1-M methanol. Reproduced from [97], with permission from American Chemical Society, 2011.

Strategy of increasing active sides for suitable absorption of light is a way to enhance photoelectrochemical performances of photocatalysts. For example, Jiao et al. demonstrated different morphology of tungsten trioxide hydrate (3WO_3_·H_2_O) films which grown by hydrothermal method using Na_2_SO_4_, (NH_4_)_2_SO_4_ and CH_3_COONH_4_ as capping agents, respectively. Platelike, wedgelike and sheet like nanostructures can be obtained as shown in Figure 8 [97]. From Figure 8e it can be seen that sheet like nanostructures had the highest photocurrent density (1.15 mA/cm^2^) under illumination and high photocatalytic activity for photodegradation of methanol. This was in good agreement with UV-vis absorbance spectroscopy results (Figure 8d). The authors believe that the reason for high current density of sheetlike morphology can be explained by the existence of small pores among sheets. This may be beneficial for accelerating the interface electron kinetics between the sheet and electrolyte due to its large active surface area.

Davidne et al. [98] reviewed WO_3_ nanostructures and studied the effect of morphology on photocatalytic activity for decomposition organic dyes. Nanostructures such as nanoplate, nanoneedle, nanorods and nanowire were obtained by hydrothermal method. It was found that photocatalytic efficiency has good correlation with band gap, crystalline phase, morphology and oxidation state. Nanoneedles with hexagonal structure showed the best photocatalytic efficiency in contrast to others.

Monoclinic nanorods showed higher photocurrent density than (2.09 mA/cm^2^) nanoplates (1.61 mA/cm^2^) in the hydrogen evolution reaction (HER) [96]. Some results concluded that 1D structures have high optical, electrical, photoconductivity properties and fast charge transportation [99,100,101]. Vertically oriented nanorods and nanoneedles have remarkable PEC results [65,66,102] due to high interfacial contact area which improves redox contact area and efficient light scattering.

However, some authors believed that 2D nanostructures like nanoplates have higher specific surface area than one-dimensional (1D) materials such as nanorods and nanowires. For example, Su and others demonstrated better photoelectrochemical characteristics and optical properties of WO_3_ nanoflakes than WO_3_ nanorods [87]. Hammad et al. [103] fabricated WO_3_ nanorods (with a diameter 7 nm, length up to 700 nm) and WO_3_ nanoplates with width 700 nm on fluorine-doped tin oxide (FTO) substrate via hydrothermal treatment. Results of electrochemical spectroscopy showed that nanoplates have better contact with substrate than nanorods which led to high photocurrent density of 400 μA/cm^2^ over 350 μA/cm^2^. Through changing concentration of HCl acid, Zhou et al. [104] synthesized perpendicularly oriented WO_3_ nanorods and nanoplates at different amount of acid. WO_3_ nanoplate arrays also showed a superior photocurrent density of 1 mA/cm^2^ at 1.6 V vs. RHE than nanorods of 0.8 mA/cm^2^. A high photocurrent density may be related with a long carrier diffusion length through 2D nanostructures comparing with 1D and efficient charge transportation and separation. Contradictory results between 2D and 1D nanostructures allow them to conclude that comparing different morphologies under different conditions do not give us true information. This is because one morphology can show different results depending on its morphologic parameter such as length, thickness and diameter.

**Figure 9:**
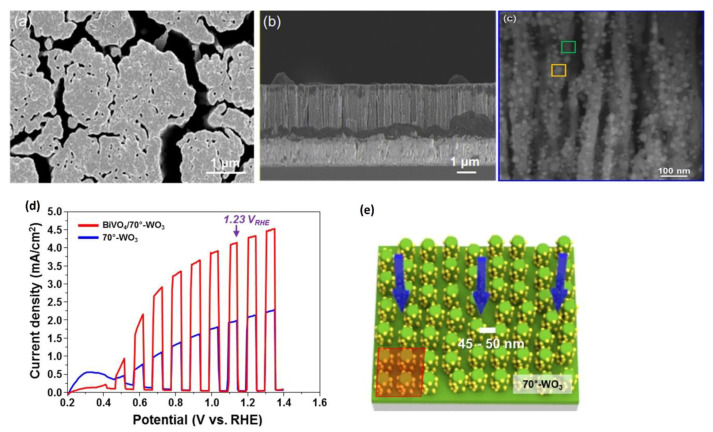
(**a**,**b**) Top and cross-section SEM images of the optimum BiVO4/70°-WO_3_ nanorods; (**c**) expanded image of BiVO_4_/WO_3_ nanorods, (**d**) enhancement of photocurrent density of BiVO_4_/WO_3_ nanorods (70°) nanorods; (**e**) schematic illustration of BiVO_4_/WO_3_ nanorods. Reproduced from [65], with permission from Elsevier, 2016.

In the aspect of photocatalytic efficiency evaluation in combination with morphology, WO_3_–BiVO_4_ nanostructures have been mostly studied. Lee and others [65] fabricated WO_3_–BiVO_4_, TiO_2_–BiVO_4_, Fe_2_O_3_–BiVO_4_ and SnO_2_–BiVO_4_ nanostructures and showed that PEC characteristics of bare WO_3_ dramatically increased after modification with BiVO_4_. The SEM and cross-sectional images of WO_3_ nanorods coated with BiVO_4_ are presented in Figure 9. They concluded that pairing WO_3_ with BiVO_4_ creates very promising photoanodes for water oxidation than others.

Improving PEC characteristics can be achieved by also using core–shell structures. Spatial separation of photogenerated charges between the core and shell is beneficial, however, excited charges stay inside and do not react with electrolyte. Nevertheless, fast transportation of charges to surface can diminish shell thickness [105]. Rao et al. [106] synthesized core–shell nanostructures of WO_3_–BiVO_4_ to improve light absorption and charge separation. A photocurrent and an incident photon-to-current conversion efficiency reached 3.1 mA/cm^2^ and ~60% at 300−450 nm, respectively at 1.23 V vs. RHE under simulated sunlight.

Enhanced PEC performance was obtained by designing yolk-shell-shaped WO_3_/BiVO_4_ heterojunction which produced a photocurrent density of 2.3 mA/cm^2^ with the highest value of ~5.0 mA/cm^2^ after adding a Fe–Ni co-catalyst at a bias of 1.23 V vs. RHE under AM 1.5 illumination (100 mW/cm^2^) [107]. These noticeable photocurrent density results demonstrated that core–shell structures may be potentially viable for photocatalytic applications.

**Figure 10:**
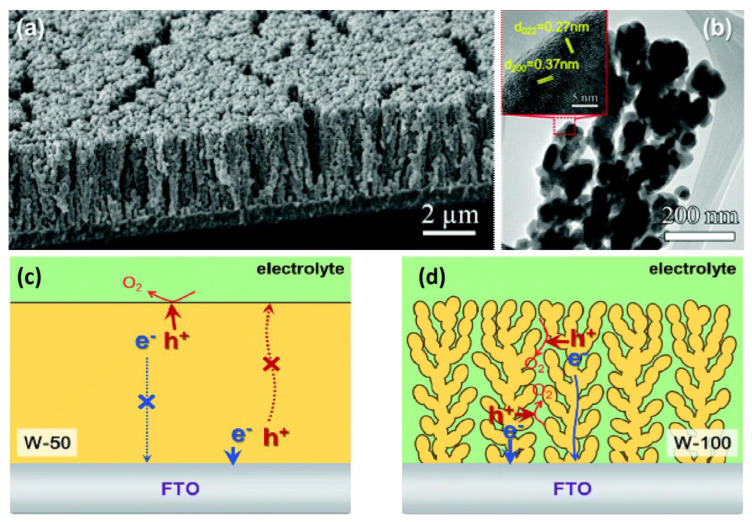
(**a**) SEM image of the WO_3_ photoanode; (**b**) TEM image of the nanoporous WO_3_ clusters, the inset: a high resolution TEM image; (**c**,**d**) schematic illustrations of charge transport/transfer processes. Reproduced from [114], with permission from Royal Society of Chemistry, 2015.

Nanostructures with nanoporosity have shown a better PEC activity due to their large specific surface areas, relatively higher light absorption rate and excellent charge collection efficiency [108,109,110]. A high surface area of porous nanostructures makes them promising electrode materials for electrochemical surface reactions [111,112,113]. Furthermore, the nanoporous structure creates the depletion layer and reduced diffusion distance to the photoelectrodes/electrolyte interface, which diminish recombination of electrons and holes [114,115,116]. Song et al. [117] used versatile foaming-assisted electrospinning method to produce mesoporous WO_3_ nanobelts which enhanced the PEC water-splitting performance compared with the as-prepared WO_3_ nanofiber and WO_3_ nanobelt samples. Shin et al. [114] used a laser ablation method to produce tree-like nanoporous WO_3_ photoanode for a photoelectrochemical water–oxidation performance. Both SEM and TEM image in Figure 10, show 1D treelike morphology with a thickness ~3.2 μm and many clusters with nanoporous with average size of ~60 nm. The photocurrent density of treelike porous structures was 9-fold higher (1.8 mA/cm^2^ at 1.23 V vs. RHE)) than dense WO_3_. A quantum efficiency (QE) or incident photon-to-electron conversion efficiency (IPCE) was 70% at 350–400 nm.

Fujimoto et al. [115] synthesized porous BiVO_4_ using the auto combustion method. Adding oxidizing agent NH_4_NO_3_ and subsequent decomposition of organic additive after heating allowed to create porous film with small crystalline BiVO_4_ nanoparticles during the synthesis. The optimized WO_3_/BiVO_4_ film produced a maximum IPCE value of 64% at 440 nm with photocurrent density of 3.43 mA cm^2^ at 1.23 V vs. RHE (under one sun illumination).

Finally, it is obvious that different morphologies as a factor of synthesis method produce different PEC results and play an important role in configuration of water-splitting devices. However, it is hard to conclude that one morphology is more beneficial than others. In fact, other factors such as substrate on which the structure is grown, electrolyte, capping agents and, etc. may be cause of change in photocatalytic activity.

### 4.3. Binary Structures of Hierarchical Architectures Based on WO_3_ Semiconductors

As mentioned above, single photocatalysts cannot satisfy all the requirements needed for water-splitting PEC systems. Therefore, scientists focus most of their attention on creating different kinds of heterostructure architectures from different various materials such as metal oxide/metal oxides [47,118,119,120], metal oxide/metals [92,121,122,123] and metal oxide/inorganic compounds [124] to create efficient systems for various spheres. Mixing several materials is a method commonly used to improve separation of charge carriers, photoelectrode stability, absorption of visible light, suitable carrier diffusion length and effective surface charge transfer.

#### 4.3.1. Metal Oxide/Metal Oxide Binary Heterostructures

The sensitivity of TiO_2_ can also be obtained by modification the surface using semiconductor photocatalyst with a smaller bandgap as WO_3_ [125,126,127]. A WO_3_/TiO_2_ photocatalytic system was published in 1998 [128]. The photocatalytic activity of WO_3_ coated with TiO_2_ was tested for the oxidation of water using iron (III) acceptor. It was also found that iron (III) ion is preferred more than iron (II) ion as an electron acceptor for oxygen evolution.

Amorphous α-TiO_2_ can be used to passivate the surface of WO_3_ based nanostructured photoanodes. For example, Yang and et al. [129] demonstrated high photocurrent with 1.4 mA/cm^2^ at 0.8 V in 0.1-M Na_2_SO_4_ electrolyte using WO_3_ nanoflakes coated amorphous α-TiO_2_ films. Passivation of WO_3_ by α-TiO_2_ was realized through the O^2−^W^6+^ bonding at contact surface between WO_3_ and α-TiO_2_. Hence, passivation of surface allows to decrease recombination and improve PEC oxidation.

Hierarchical WO_3_/TiO_2_ composites for hydrogen evolution was fabricated by Momeni [130] using the anodization method. TiO_2_ nanotubes with a diameter ranged 80–110 nm were modified by WO_3_. Controlling the concentration of the Na_2_WO_4_ solution allowed them to achieve the highest amount of H_2_, with 2.14 mL/cm^2^ under 120 min of solar illumination, which is approximately 3.02-fold higher than bare samples with TiO_2_ nanotubes (0.71 mL/cm^2^). It also showed increase of photocurrent value from 0.81 to 1.61 mA/cm^2^ after modification proved the effectiveness of the coupled WO_3_/TiO_2_ system. The anodization method was also successfully used to prepare the hybrid WO_3_/TiO_2_ nanotube photoelectrodes [131] which showed better photo conversion efficiency, STH efficiency and H_2_ generation.

Many other studies highlighted that coupled WO_3_–TiO_2_ systems have better characteristics. For example, the highest photocatalytic activity of nanocomposites particles for degradation of Rhodamine B [60,61], decomposition of 1,4-dichlorobenzene (DCB) aqueous solution [132] and azo dyes [133], for effective catalytic oxidation cyclopentene to glutaraldehyde [133] were obtained.

The effectiveness of photocatalysts was also attained by engineering morphology and specific surface area of the material since electron-hole transfer occurs on the surface. According to some studies, although electron–hole pairs can be generated in volume, they can annihilate before they reach the surface due to the low diffusion length.

**Figure 11:**
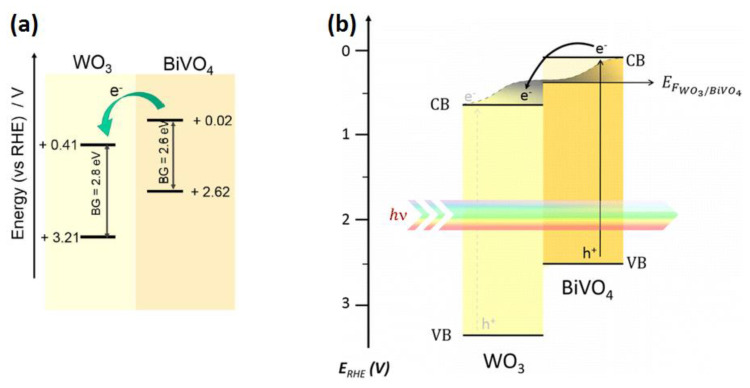
(**a**) Diagram of the band edge positions of pure WO_3_ and BiVO_4_ and (**b**) for a WO_3_–BiVO_4_ composite under solar irradiation. Reproduced from [137], with permission from American Chemical Society, 2015.

Most of authors have shown that dual heterostructures of WO_3_ and BiVO_4_ are effective for driving water oxidation reactions [64,134,135]. BiVO_4_ is an n-type semiconductor-like WO_3_ which has a bandgap around 2.4 eV [26,136]. The theoretical solar-to-hydrogen efficiency using this material (9.8%) is more than that of WO_3_ (4.8%)-based systems. Despite the fact that BiVO_4_ is a direct semiconductor unlike WO_3_, it has a poor charge transport and a short hole-diffusion length (~60 nm). Coupling WO_3_ with BiVO_4_ decreases recombination of photogenerated charge carriers and improves the efficiency of overall water-splitting systems. A dynamic of photogenerated carriers and effective charge separation of WO_3_–BiVO_4_ heterojunctions was explained by Grigioni using femtosecond transient absorption spectroscopy [137]. They determined the position of the WO_3_ conduction bands (+0.41 V vs. RHE) and BiVO_4_ (+0.02 V vs. RHE) by testing the photocatalytic reduction of thionine. The charge separation mechanism of BiVO_4_–WO_3_ system is shown in Figure 11a. When comparing the flat band position of BiVO_4_ in the WO_3_–BiVO_4_ composite, a shift of 170 mV is observed. This shift was explained by electron equilibrium between the two materials due to a Fermi level shift. Photoelectrons transfer from BiVO_4_ to WO_3_ while holes localize in BiVO_4_, so it is possible to separate photogenerated charge carriers spatially (Figure 11b).

The morphology of WO_3_ and the decoration method with BiVO_4_ are also very important issues. Chae et al. synthesized mesoporous WO_3_ films followed by a coating of BiVO_4_ to research particle sizes and shapes, as well as the effects of the photoanode thickness. Large nanoplates showed a high injection efficiency while nanospheres enhanced the charge-separation efficiency [138]. Pihosh et al. synthesized WO_3_–BiVO_4_ vertically oriented nanorods by combining the glancing-angle deposition and normal physical sputtering techniques [66]. The photocurrent density achieved 3.1 mA/cm^2^ at 1.23 V RHE under illumination of one sun. A nanopillar morphology of WO_3_–BiVO_4_ photoanodes fabricated by electrostatic spraying method also produced a photocurrent up to 3.2 mA/cm^2^ [139]. An enhanced photocurrent density of 4.55 mA/cm^2^ was achieved by using a WO_3_–BiVO_4_ photoanode [65]. Deposition of BiVO_4_ nanodots on WO_3_ nanorods had an increased photon to hydrogen efficiency of 80% at 1.23 V vs. RHE, which is higher than the theoretical efficiency for bare BiVO_4_. Rao et al. fabricated WO_3_–BiVO_4_ core shell nanowires and showed that the photoanodes demonstrated a $\eta_{arb}\times \eta_{sep}$ up to 53%. A combination of BiVO_4_ with more conductive WO_3_ leads to effective charge carrier separation and the photocurrent achieved 3.1 mA/cm^2^ at 1.23 V vs. RHE.

**Figure 12:**
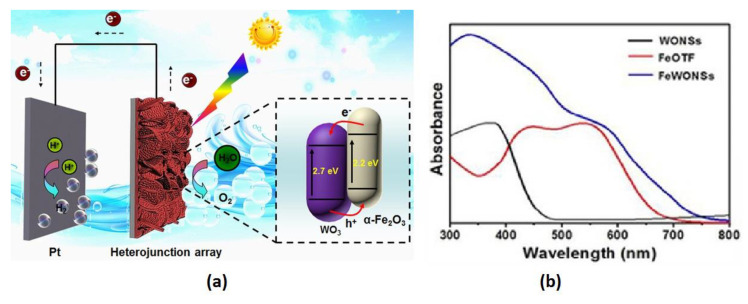
(**a**) WO_3_–α–Fe_2_O_3_ nanocomposites and its band diagram for PEC water-splitting (**b**) UV-vis absorption spectra of WO_3_@Fe_2_O_3_. Reproduced from ref [142], with permission from John Wiley and Sons, 2016.

Iron is an abundant and important metal in the earth’s crust, so its use is considered economically viable. Oxidation of iron can lead to formation of the known hematite phase α-Fe_2_O_3_ which has semiconductor properties. It has good stability in most electrolytes pH > 3 and has a narrow bandgap (~2.2 eV) which can absorb 40% of the solar spectrum. Although hematite electrodes are well studied for PEC system, photoconversion efficiency is still lower than the theoretical value due to low hole mobility (~2–4 nm). Moreover, poor electrical conductivity, high recombination rate of electron-hole pairs [140] and the slow kinetics of oxygen evolution [141] limits its use. Some studies focus on binary heterostructures with WO_3_–hematite α-Fe_2_O_3_ photoanodes [142]. A photocurrent of 1.66 mV/cm^2^ was observed at 1.23 V RHE, while the photon to current efficiency was 73.7% at 390 nm. Schematic illustration of WO_3_-Fe_2_O_3_ composite nanosheets and bandgaps are shown in Figure 12a. The optical absorption measured by a UV-vis diffuse reflectance spectroscopy was found to be improved for the composite WO_3_ and Fe_2_O_3_ material (Figure 12b). Luo published enhanced electrochemical characteristics of a WO_3_@Fe_2_O_3_ photoelectrode compared to bare WO_3_ and Fe_2_O_3_ [143]. Effective photoelectrochemical splitting of seawater with Fe_2_O_3_/WO_3_ nanorods was achieved by Li et al. [144]. Although optical absorption is promising, the photocurrent of Fe_2_O_3_/WO_3_-based photoanodes is still low.

#### 4.3.2. Metaloxide/Inorganic Compounds Heterostructures

The heterostructures formed from WO_3_ and sulfur components have narrow bandgaps. For example, antimony sulfide, Sb_2_S_3_, (1.7–1.9 eV)_,_ bismuth sulfite, Bi_2_S_3_, (~1.3 eV) and tungsten disulfide, WS_2_, (~1.3 eV) [145] are very effective. For example, Zhang [146] synthesized WO_3_/Sb_2_S_3_ heterostructures via a simple hydrothermal method to improve PEC performances. Tungsten trioxide nanorods and nanoplates were synthesized by controlling the concentration of acid and tungsten precursor along with subsequent growth of Sb_2_S_3_ nanoparticles. It was demonstrated, that WO_3_/Sb_2_S_3_ heterostructures have better electrochemical and optical characteristics than pristine WO_3_.

A high photocurrent of 5.95 mV/cm^2^ was achieved using a three-dimensional WO_3_/Bi_2_S_3_ heterojunction [147]. Bi_2_S_3_ is also a n-type semiconductor with bandgap 1.3 eV and has more negative conduction band edge than WO_3_. A WO_3_/Bi_2_S_3_ heterojunction was fabricated by combining of hydrothermal method, SILAR (successive ionic layer absorption and reaction) process and CBD (chemical bath deposition). Relatively high light absorption, small electron transfer impedance and high charge carrier were proved by UV-vis, EIS (Electrochemical Impedance Spectroscopy) and Mott-Shottky methods.

Despite the fact that the WO_3_ bandgap energy is not suitable for hydrogen evolution, it is still useful for solving problems such as high electron-hole recombination rates and poor electrical conductivity of some photocathodes [145]. A study of WO_3_@WS_2_ core–shell nanostructures fabricated by plasma assisted sublimation was published by Kumar et al. [145]. The highest achieved cathodic photocurrent was 16.2 mA/cm^2^ for WS_2_ at 0.3 V vs. RHE. Sulfurization of the WO_3_ surface forms a WS_2_ layer with a rich defect structure, resulting in a high catalytic activity.

#### 4.3.3. Metal Oxide/Plasmon Particle Systems

**Figure 13:**
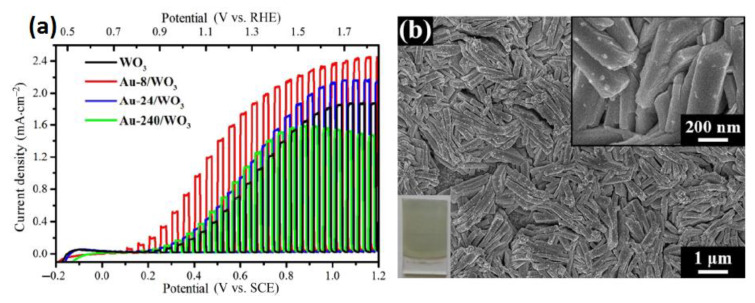
(**a**) Current -potential curves of WO_3_@Au composite with HAuCl_4_ concentrations of 8, 24 and 240 umol in 0.1-M Na_2_SO_4_ electrolyte and (**b**) SEM images of WO_3_@Au composites. Reproduced from ref [149] with permission from Springer Nature, 2016.

The plasmonic effect induced by noble metal particles plays an important role in decorating photoelectrodes. Photoactivity of photocatalysts can be increased by enhancing light scattering and SPR [148,149]. Moreover, noble metals play a role as a co-catalyst for OER due to good electrical contact between the metal and semiconductor [150,151]. Altering the surface properties of WO_3_ photoanodes with plasmonic nanoparticles Au and Ag has shown enhanced visible light absorption and high photocurrent density [152]. Hu showed a high faradic efficiency of 94% for WO_3_@Au composites [149]. Enhanced photocurrent density and morphology of heterostructure is presented in Figure 13a,b. Modified WO_3_ by plasmonic Ag and Pt nanoparticles showed enhanced photocurrent of 1.13 mA/cm^2^ at 1.23 V vs. RHE under AM 1.5G illumination in a 0.2 M Na_2_SO_4_ solution, which is nearly 3.32 times that of bare WO_3_ [153]. The photocurrent density for binary systems are represented in Table 1.

### 4.4. Ternary Systems for Efficient Water Decomposition

**Figure 14:**
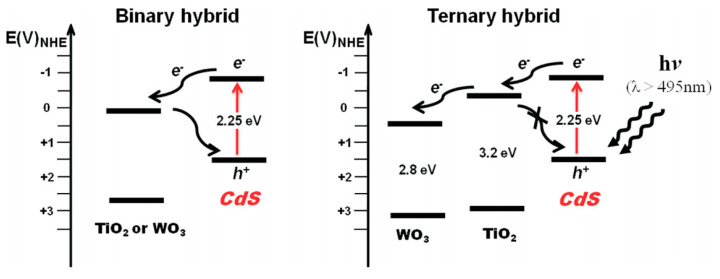
Schematic representation of a comparison of electronic transition processes in binary and ternary hybrid. Reproduced from [156], with permission from American Chemical Society, 2011.

A typical heterojunction between two dissimilar semiconductors comes to equilibrium without any external electric field. The result shown above is a potential difference that appears at the interface as an internal electric field. This field accelerates charge carrier drift and decreases the number of electron–hole recombination, improving the semiconductor’s photocatalytic activity [155]. The exploitation of multilayer structures in photocatalysis is considered more beneficial. An illustration of the advantages of a cascade transition of charge carriers is well explained in numerous works [156] and [157]. The authors of [156] work investigated the photocatalytic properties of a composite of CdS, TiO_2_ and tungsten trioxide. Since, TiO_2_ has the conduction band edge which is between conduction band edges of CdS and WO_3_, in such ternary composites electronic transitions are cascading. After generation of excitons in CdS, electrons easily migrate to TiO_2_ and WO_3_ of potential difference as shown in Figure 14.

In addition to ternary composites made only of semiconductor materials, there are also ternary structures based on semiconductors and metal complexes. An example of such ternary photocatalysts can be photonic heterostructure of CdS–Au–WO_3_. Cui et al. reported, that Au nanoparticles deposited between WO_3_ and CdS leads to form heterostructure which had photocatalytic properties superior to similar two-phase systems. It was thought that such amelioration caused by a synergistic integration of photon absorption with effective electron transfer in the heterostructure [62]. The use of doping elements—or modification by particles such as CdS quantum dots [158] in usual two-component heterojunctions—is also referred to as a ternary structure. Doping elements such as Yb [159] and Mo have been shown to suppress charge carrier recombination during photocatalysis [160], improving the efficiency of reactions. In ref. [161], high photocurrent density was achieved by doping active materials to make bilayers WO_3_/MoBiVO_4_ (BiV_0.95_Mo_0.05_O_4_). The photocurrent of the Mo-doped content increased by a factor of 3 and 1.5 relative to pristine photoanodes based on WO_3_ and BiVO_4_/WO_3_, respectively. Another promising way to create performable photocatalysts is a combination of a catalyst with heterostructures. Some examples of such formations are materials which obtained by the deposition of catalytic coatings NiOOH [162] and FeOOH [163] on the surface of two-phase WO_3_ structures. The deposited materials suppress both the degradation of the photoactive material and the Faradic losses [164], accelerating the photoelectrochemical reaction processes. Shouli Bai et al. also combined catalyst layers with a heterojunction by depositing NiFe bimetallic complexes onto a WO_3_/Fe_2_O_3_ surface [165]. Their strategy increased the photocurrent density of the ternary photoanode system to 3.0 mA cm^−2^, which, according to Shouli Bai, is 5 and 7-fold higher relative to that of pristine WO_3_ and α-Fe_2_O_3_ structures, respectively. The role of the catalyst here is to improve the absorption of holes accumulated on the electrode surface. In another study, the deposition of CoPd bimetallic nanoparticles onto the surface of WO_3_/α-Fe_2_O_3_ photoanodes causes a cathode shift of the initial potential, increasing the photocurrent density from 0.15 to 0.5 mA/cm^2^ during water oxidation at 1.23 V relative to RHE when illuminated with AM 1.5 G [166]. Substitution of iron oxide with cadmium sulfide in heterostructures based on WO_3_ also makes it possible to sensitively increase the photoresponse of electrodes during water decomposition. In [167] work preparation of such ternary compositions conducted via three simple hydrothermal, impregnation and photo-assisted deposition steps. Thus, authors obtained rodlike structures with a performance of photocurrent at the level of 5.85 mA/cm^2^ at 1.23 V (vs. RHE). Sun with co-workers explain this phenomenon by creation a larger built-in potential at interface WO_3_/CdS formed via impregnating appropriate CdS onto surface of WO_3_. This drives transport of electrons from CdS to WO_3_ with improvement of exciton separation. In this case, not the entire charge is torn well enough. Part of the charge recombines due to the weak involvement of holes in the valence band of cadmium sulfide in the process of water oxidation. Decoration surface of WO_3_/CdS heterojunctions with Co-Pi co-catalyst advances the transfer kinetics of charge advanced which is positive to suppression of charge recombination. In this case, the mechanism of improving charge transfer to the sites of redox half-reactions is also achieved by adding phosphate anions to the electrolyte. In fact, the use of various electrolytes, such as glycerol-water mixture [168], in its effect on the parameters of the transferred charges between photocatalytic coatings and a split liquid. Varieties of compositions of working electrolytes and a list of structural heterojunctions, as well as geometric schemes that receive the influence and influence of all this on the photoresponse of PEC systems are shown in Table 2. It is known that tungsten trioxide is widely used as the primary semiconductor material in three transient systems in photocatalysis. Therefore, numerous of recent works dedicated to photoinduced dye degradation processes [169] and the direct splitting of water [170,171] were carried out with exploiting WO_3_. Liu formed an effective three-component photoanode based on tungsten trioxide nanosheets synthesized by hydrothermal method and decorated with Zn_x_Bi_2_S_3+x_ quantum dots via layer-by-layer adsorption [172]. In obtained core/shell structure of Zn_x_Bi_2_S_3+x_/WO_3_, surface Zn_x_Bi_2_S_3+x_ served as a protective layer for tungsten trioxide. Comparable photocatalytic studies conducted under visible light irradiation in 0.1-M Na_2_S and 0.1-M Na_2_SO_3_ aqueous solution at pH ~9 showed that Zn_x_Bi_2_S_3+x_/WO_3_ composite has higher photocatalytic activity then Bi_2_S_3_/WO_3_.structure. The photocurrent density was 7 mA/cm^2^ at a bias voltage of −0.1 V. This attributed to the involvement of ZnS nanostructures with high photocatalytic properties [173]. Designing semiconducting heterocomposites via a surface–interface engineering approach showed high effectiveness for enhancing exciton separation/transportability and photoelectrochemical features. The photoactive layer of 2D BiVO_4_-layer/WO_3_ array modified with cobalt phosphate studied for oxygen evolution and showed 1.8 mA/cm^2^ at 1.23 V vs. RHE in a phosphate buffer under an AM1.5G sun. The result is 5 and 12-fold higher than that of bare WO_3_ and BiVO_4_ photoanodes, respectively [174]. Morphology of WO_3_ nanostructures affects to charge separation ability in the active layer and to charge collection efficiency in the WO_3_/BiVO_4_ heterojunction. The low-dimensional nanosphere WO_3_ layer showed higher photocatalytic efficiency than the platelike or rodlike ones [71].

There are three different component systems with different morphology in which the hierarchy of the band structure observed for effective water-splitting. The main types of morphology include multi-heterojunction-based photocatalysts composed of WO_3_ nanorods, Pt nanoparticles and TiO_2_ nanoparticles [175], hierarchical heterostructures with core and double shells [176], rectangular AgIn(WO_4_)_2_ nanotubes which showed excellent photocatalytic properties for decomposing water to evolve H_2_ [177] and linked porous structures such as WO_3_/porous–BiVO_4_/FeOOH [178]. Special attention should be paid to the three-component system made on spiral WO_3_ nanostructures decorated with doped Mo and BiVO_4_ nanoparticles [179]. Based on the assertions that 1D structures charge carriers go straight to the terminals or half-reaction centers [180], Xinjian Shi et al. used a spring morphology with an increased surface area while retaining the properties of 1D structures. As a result of the successive deposition of these structures onto conductive glass, a spiral heterocomposite WO_3_/(W, Mo)–BiVO_4_ with a length of 5.5 µm was obtained and the photocurrent density exceeded 3.9 mA/cm^2^. The process of making triple transitions is possible using various techniques, such as electrochemical reduction-oxidation technology [181], a hybrid synthetic method, including electrodeposition and hydrothermal treatment [182], a solvothermal-calcination process [183], an electrostatic-driven self-assembly correlated with ion-exchange process [162] and a polymer complex method [184]. Jun Lv et al. obtained photoactive LiCr(WO_4_)_2_. After heat treatment at 700 ℃ for 5 h, crystal structures of tungstate were obtained, consisting of alternating layers of WO_6_ and CrO_6_ octahedra lying on the YZ plane. The WO_6_ octahedra are connected by separating edges, leading to the appearance of unrelated zigzag rows along the *Y*-axis. On the other hand, CrO_6_ octahedra not related to each other. Photocatalytic tests of LiCr(WO_4_)_2_ showed that the release of H_2_ proceeds upon irradiation with visible light up to 540 nm [184]. Reaching the rest of the visible spectrum remains the goal. Ji Hyun Baek et al. developed a photoanode based on BiVO_4_/WO_3_/SnO_2_ using a simple method of applying layers on conductive glass to obtain a thin double-heterojunction coating on the order of 320 nm. A characteristic feature of BiVO_4_/WO_3_/SnO_2_ is the large bandwidth of visible light, which allowed the researchers to combine the photoanode with a perovskite solar cell into a tandem PEC system. This allowed the reaction to split water without applying a bias voltage [26]. In general, the development of PEC cells using independent absorbents of incident light is a promising direction, so the next section will deal tandem hydrogen production systems.

### 4.5. WO_3_-Based Tandem PEC Cells

Two strategies can produce tandem cells for photoinduced water-splitting. One strategy used is to increase the capture of photons is a tandem system of a photoanode and photocathode with n- and p-conductivities of active layers, respectively. The splitting of water under light irradiation in this type of PEC cell occurs as a result water oxidation by photogenerated holes on the photoanode surface. Meanwhile, reduction to molecular hydrogen is initiated by electrons on the photocathode surface. At the same time, negative charge carriers generated in the photoanode are directed under the action of the field, to positively charged carriers in the photocathode material, where they recombine. Robert Coridan et al. investigated the photocatalytic properties of Si/WO_3_ heterojunctions and Si/ITO/WO_3_ arrays in a core-shell manner [185]. The operation of the tandem structure depends not only on the bandgap of the semiconductors used but also on the alignment of the strip edge and the state of the electrical connection between photo absorbents. When a mismatch of conductivity levels and valence levels of semiconductors included in tandem circuits occurs, low efficiencies of PEC hydrogen evolution cells [186]. A similar effect was observed in the work of Heli Wang et al. in which they combined n-type tungsten trioxide and hematite nanorod metal oxides with p-GaInP_2_. It was found that even when photo electrodes are illuminated with a source with a power of 1 W/cm^2^, a photocurrent appears but the density values of which are rather weak. This is due to low electron mobility of in the hematite layers, short hole-diffusion length, and insufficient potential difference between the levels of the conduction band and valence semiconductors, which help reduce charge recombination [187]. Geometrical optimization of the morphology of the active layers of photoelectrodes refers to an increase the density of short circuit current [188]. Investigating the PEC properties of the tandem structure of WO_3_/Si, Zhuo Xing et al. concluded that it was necessary to add an intermediate layer between p and n semiconductors to reduce the number of recombinations of photoinduced charges. In [189], metal W served as an intermediate layer, resulting in a WO_3_/W/Si ternary structure demonstrated an increase in the photogenerated current density by a factor of 10 compared to the WO_3_/Si structure.

Another possible way for general water-splitting without assistance is to combine photoelectrodes with photovoltaic cells to form a tandem PEC/PV cell. In one study [190], organic molecules were used as sensitizers in a tandem PEC, which is a powerful strategy for designing hydrogen evolution systems since they allow large-scale modification of photoelectrodes by adjusting the dye redox potentials or redox mediators.

**Figure 15:**
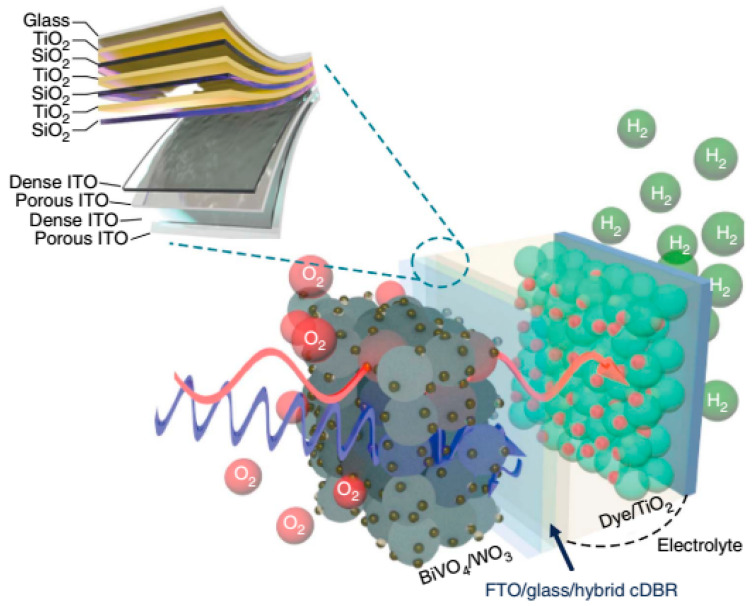
Schematic illustration of a PEC tandem system based on BiVO_4_/WO_3_ and DSSC. Reproduced from [191], with permission from Springer Nature, 2016.

In the tandem devices shown in Figure 15, the BiVO_4_/WO_3_ photoelectrode absorbs short-wavelength photons and more extended wavelength absorbs by a dye/TiO_2_ electrode [185,186,187,188,189,190]. This method offers better concession between device performance, complexity and stability [3]. In addition to scientific methods, when choosing materials, morphology and hierarchy of architecture, engineering aspects related to the spatial and reciprocal arrangement of the physical elements of tandem structures are also important. For efficient use of incident photons, Pihosh Y. and colleagues produced a PEC-PV tandem system based on WO_3_ NRs/BiVO_4_+CoPi photoanode and an AlGaAsP solar cell, which were placed at 45° relative to each other using a V-shaped stand [102]. This design allows the passage of reflected light from the photoanode to the surface of the solar cell.

Thus, the characteristic differences between ternary systems and binary systems are the improvement of photocatalytic properties and corrosion resistance. As described above, tandem structures provide an operating mode for photoelectrochemical processes in a wide range of the radiation spectrum. It increases the number of components of the hydrogen evolution cells, which leads to a complication of the assembly of heterostructures and to a high cost of the obtained layers. Therefore, when choosing which or used components of tertiary composites, pay attention to postprocessing, including thermal.

## 5. Conclusions and Outlook

The goal of research in the field of photoinduced decomposition of water is to develop high performance photocatalytic systems with high STH efficiency. The transmission of the photocatalytic systems from the field of laboratory research to the large scale production is a key point. The principle of using semiconductor coatings based on tungsten trioxide for PEC cells is justified by the economic aspects associated with the low cost of the material, as well as with its physicochemical properties. Using nanotechnology and nanomaterials is a suitable method for addressing several of the issues listed above. Metal oxide nanoparticles can be obtained by a wide range of physical and chemical methods. They can be classified as top-down and bottom-up methods. Top-down approaches rely on physical processes, such as abrasion or ball milling. Nano powders produced in this method usually exhibit wide distribution sizes and their size, shape and morphology are difficult to control. In addition, possible structural and surface impurities can have a significant effect on surface chemistry and the catalytic properties of nanomaterials. Low-dimensional structures are most advantageous from the point of view of effective absorption of light with the generation of charge carriers, migration of charge carriers to the surfaces of the material, which fit over the exciton lifetime, as well as possessing a fairly significant semiconductor/electrolyte contact area. The mixing of semiconductors (i.e., the formation of composites) is also an accepted strategy for the development of photocatalysts that respond to radiation in the visible range. This strategy is based on a hierarchical architecture for connecting a wide-gap semiconductor with a narrow-gap semiconductor with a more negative level of the conduction band. Thus, the conduction of electrons can be introduced from a narrow-gap semiconductor into a wide band semiconductor, leading to better absorption for the mixed photocatalyst. An additional advantage of using composite semiconductor photocatalysts are that it reduces carrier recombination by facilitating electron transfer crossing interface of particles. In photocatalyst composites, semiconductor particles stay in electronic contact individually. For a successful combination of semiconductors, the certain requirements are needed to be met: the conductivity level of the narrow-gap semiconductor should be more negative than the level of the wide-gap semiconductor; the position of the conductivity level of the wide-gap semiconductor should be more negative than the recovery potential; electron injection should be quick. All of the steps can improve the characteristics of the material, as well as eliminate the influence of its shortcomings on the process of splitting water under the action of light. In any case, a review of the literature in this area indicates a special level of development in the field of photoelectrochemistry for hydrogen evolution using active materials from pure tungsten trioxide or in various compositions with it. However, so far, the complete and qualitative decomposition of water and the generation of hydrogen under the influence of sunlight has a low rate, which indicates insufficient feasibility of industrial use of existing technologies. Based on the current trend towards the creative and experimental activity of researchers in this direction, the authors of this article express deep confidence in the imminent achievement of quantum efficiency of PEC systems sufficient for universal use in human life in the near future.

## Figures and Tables

**Table 1:** Photocurrent densities of binary heterostructures.

Photoanodes	Methods	Morphology	Electrolyte	Potential	P (mW/cm^2^)	J (mA/cm^2^)/STH (%)/Gas Evolution (mL/cm^2^)	Ref
WO_3_/TiO_2_	Solvothermal	Nanoflake	0.1-M Na_2_SO_4_	0.8 vs. SCE (1.45 vs. RHE)	100	1.4	[129]
WO_3_/TiO_2_	Anodization	Nanotubes	1-M NaOH	0.7 vs. Ag/AgCl (1.7 RHE)	100	1.6	[130]
WO_3_/TiO_2_	Anodization	Nanotubes	1-M KOH	0.6 V vs. SCE (1.62 RHE)	100	2 /3.1%/16.2	[131]
WO_3−x_/ZnO	Solvothermal method	Nanorods	1-M Na_2_SO_4_	1.23 vs. RHE	100	3.38	[56]
WO_3_/BiVO_4_	Glancing-angle deposition/electrodeposition	Vertically oriented nanorods	0.5-M Na_2_SO_4_	1.23 vs. RHE	100	3.1	[66]
WO_3_/BiVO_4_	Electrostaticspraying method	Nanotextured pillar	0.5-M Na_2_SO_4_	0.7 V vs. Ag/AgCl (1.44 vs. RHE)	100	2.1	[139]
WO_3_/BiVO_4_	Layer-by-layer	Film	0.5-M Na_2_SO_4_	1.23 vs. Ag/AgCl (1.817 vs. RHE)	100	2.78	[134]
WO_3_/BiVO_4_	Spin coating	Film	0.5-M Na_2_SO_4_	1.23 V vs. Ag/AgCl 1.817 vs. RHE	100	1.2	[64]
WO_3_/BiVO_4_	Pulsed electrodeposition	Nanorods	0.1-M Na_2_SO_3_	1.23 vs. RHE	100	4.55	[65]
WO_3_/BiVO_4_	Anodic oxidation	Nanoporous film	0.1-M KH_2_PO_4_	0.6 V vs. Ag/AgCl (1.21 vs. RHE)	100	02.01	[135]
WO_3_/BiVO_4_	Electrospinning	Nanofibers	0.5-M Na_2_SO_4_	1.23 vs. RHE	100	2.8	[154]
WO_3_/Fe_2_O_3_	Solvothermal	Nanosheets	0.5-M Na_2_SO_4_	1.23 vs. RHE	100	1.66	[142]
WO_3_/Fe_2_O_3_	Hydrothermal	Nanorods	0.1-M Na_2_SO_4_	1.23 vs. RHE	100	1	[144]
WO_3_/Fe_2_O_3_	Sol–gel	Film	0.1-M NaOH	1.23 vs. RHE	100	0.7	[67]
WO_3_/Sb_2_S_3_	Hydrothermal	Nanoplate/nanorods	1-M H_2_SO_4_	0.8 vs. RHE	100	1.79	[146]
WO_3_/Bi_2_S_3_	CBD	Nanorods/nanoplates	0.1-M Na_2_SO_3_	0.9 vs. RHE	100	5.95	[147]
WO_3_/Au	Hydrothermal	Nanoplate	0.1-M Na_2_SO_4_	1.23 vs. RHE	100	1.5	[149]

**Table 2:** Photocurrent densities of ternary heterostructures.

Photocatalytic Material	Methods	Morphology	Electrolyte	Potential	Irradiation	Photocurrent Density	Ref
WO_3_–Pt–CdS	Combination of wet-chemical, photodeposition and hydrothermal techniques	Hollow microspheres composed of small crystallites	0.5 M Na_2_SO_4_	0.5 V vs. Ag/AgCl (1.82 V vs. RHE)	Vis light	0.16 μA/cm^2^	[192]
SnO_2_/WO_3_/BiVO_4_	Combination of electron beam deposition and metal organic decomposition technique	Planar film	0.5-M Na_2_SO_3_	1.23 vs. RHE	100 mW/cm^2^	2.01 mA/cm^2^	[193]
WO_3_/C_3_N _4_//CoO_x_	Combination of a hydrothermal method with wet impregnation	film		1.23 V vs. Ag/AgCl (1.8 V vs. RHE)	100 mW/cm^2^	5.76 mA/cm^2^	[170]
CuWO_4_−WO_3_	electrodeposition	film	0.1-M KH_2_PO_4_	0.618 V vs. Ag/AgCl (1.23 vs. RHE)	100 mW/cm^2^	0.3 mA/cm^2^	[194]
WO_3_/(Er, W):BiVO_4_	spray coating	monoclinic clinobisvanite structure	0.1-M K_2_HPO_4_	1.23 V vs. RHE	100 mW/cm^2^	4.1 ± 0.19 mA cm^−2^	[195]
WO_3_/(Er, W):BiVO_4_	spray coating	monoclinic clinobisvanite structure	0.1-M K_2_HPO_4_	2.3 V vs. RHE	100 mW/cm^2^	7.2 ± 0.39 mA cm^−2^	[195]
TiO_2_/WO_3_/BiVO_4_	hydrothermal	brochosomes-like	0.5-M Na_2_SO_4_	0.35 V vs. RHE	100 mW/cm^2^	3.13 mA cm^−2^	[196]
WO_3_/ Fe_2_O_3_/Co(OH)	electrospray deposition	worm-like nanobars	0.1-M NaOH	1.23 vs. RHE		0.62 mA cm^−2^	[197]
Ag-functionalized CuWO_4_/WO_3_	electrophoretic deposition	thin film	potassium phosphate buffer solution	0.62 V vs. Ag/AgCl (1.23 V vs. RHE)		0.205 mA cm^−2^	[198]
CuWO_4_/BiVO_4_ with Co-Pi	drop-casting and thermal annealing method	nanoflakes	1.0 M of Na_2_SO_4_ with 0.1 M of sodium phosphate buffer (pH = 7)	1.23 V vs. RHE	100 mW/cm^2^	2.25 mA cm^−2^	[199]
BiVO_4_/WO_3_/SnO_2_ connected with perovskite solar cell tandem device	Spin-coating	triple-layer planar film	pH 7 phosphate buffer electrolyte	1.23 V vs. RHE	100 mW/cm^2^	3.1 mA/cm^2^	[26]
ZnWO_4_/WO_3_	Piezo-dispensing	Spot Arrays	0.1-M Na_2_SO_4_ at pH 7	0.7 V vs. Ag/AgCl (1.31 V vs. RHE)		0.75 mA/cm^2^	[200]
b-Cu_2_V_2_O_7_/WO_3_	Seeded-growth approach		0.1-M sodium borate buffer (pH 8.2) containing 0.1-M Na_2_SO_3_	1.23 V vs. RHE	100 mW/cm^2^	0.45 mA cm^−2^	[201]
CaMn_2_O_4_/WO_3_	Spin-coating	Thin film	0.5-M Na_2_SO_4_ solution (pH 6)	1.09 V vs. RHE		1.5 × 10^−3^ mA cm^−2^	[202]
Pt/WO_3_/Ag	Hydrothermal method, chemical bath, photoassisted electrodeposition	Nanorods			100 mW/cm^2^	1.13 mA/cm^2^	[153]
WO_3_/CdS/NiOOH	hydrothermal method, successive ionic layer adsorption and reaction, photo-assisted electrodeposition	Nanorods	d 0.2-M Na_2_SO_4_-0.1-M Na_2_SO_3_	1.23 V vs. RHE		1.5–2 mA/cm^2^	[203]
ZnWO_4_/WO_3_	hydrothermal	Nanorods	0.5 M Na_2_SO_4_	1.23 V vs. RHE	100 mW/cm^2^	1.87 mA cm^−2^	[204]
WO_3_/BiVO_4_/ZnO	drop-casting method, atomic layer deposition	Nanosheets	0.5-M Na_2_SO_4_	1.23 V vs. RHE	100 mW/cm^2^	2.5–3.00 mA cm^−2^	[205]
Au-surface/BiVO_4_/WO_3_/Au-bottom	hydrothermal, sol–gel spin-coated,	Nanospheres	0.5 M Na_2_SO_4_	1.23 V vs. RHE		1.31 mA/cm^2^	[63]
WO_3_/C@CoO	hydrothermal process and immersion method	Octopus tentacles-like	1.0-M KOH	55 mV (vs. RHE)		10 mA cm^−2^	[206]
WO_3_@ZnWO_4_@ZnO	layer deposition technique and hydrothermal process	nanosheets	mixed aqueous solution of 0.35-M Na2S and 0.25-M NaSO3 (pH = 13.4)	1.23 V vs. RHE	100 mW/cm^2^	~1.57 mA/cm^2^	[207]
WO_3_/rGO/Sb_2_S_3_	chemical bath deposition	nanoplates	0.5-M Na_2_SO_4_ (pH~7)	1.23 V vs. RHE		1.20 mA/cm^2^	[208]
Cu_2_O/CuO/WO_3_	Electrodeposition, spin-coating	Thin film		0 V vs. RHE		−1.9 mA/cm^2^	[209]
WO3/BiVO4/Co-Pi	Electrodeposition	composite inverse opals	0.5-M Na_2_SO_4_	1.4 V versus Ag/AgCl (0.67 V vs. RHE)	100 mW cm^−2^	4.5 mA cm^−2^	[210]
WO_3_/BiVO_4_/TiO_2_	Spin-coating, wet chemistry	platelike	0.1-M Na_2_SO_4_	1.23 V vs. RHE	100 mW/cm^2^	~3.9 mA/cm^2^	[211]
TiO_2_/WO_3_/Pt	Electrospinning technique	fibers	0.2-M Na_2_SO_4_			15–20×10^−3^ mA/cm^2^	[212]
TiO_2_-TiCl_4_-WO_3_	Hydrothermal method + Electrodeposition	nanorods	KOH	1.23 V vs NHE	100 mW/cm^2^	3.86 mW/cm^2^	[213]

## References

[1] Gratzel M. (2001). Photoelectrochemical cells. Nature.

[2] Sathre R., Scown C.D., Morrow W.R., Stevens J.C., Sharp I.D., Ager J.W., Walczak K., Houle F.A., Greenblatt J.B. (2014). Life-cycle net energy assessment of large-scale hydrogen production via photoelectrochemical water splitting. Energy Environ. Sci..

[3] Prevot M.S., Sivula K. (2013). Photoelectrochemical tandem cells for solar water splitting. J. Phys. Chem. C.

[4] Wang G., Wang H., Ling Y., Tang Y., Yang X., Fitzmorris R.C., Wang C., Zhang J.Z., Li Y. (2011). Hydrogen-treated TiO_2_ nanowire arrays for photoelectrochemical water splitting. Nano Lett..

[5] Zhang Z., Zhang L., Hedhili M.N., Zhang H., Wang P. (2012). Plasmonic gold nanocrystals coupled with photonic crystal seamlessly on TiO_2_ nanotube photoelectrodes for efficient visible light photoelectrochemical water splitting. Nano Lett..

[6] Zhang Z., Hossain M.F., Takahashi T. (2010). Photoelectrochemical water splitting on highly smooth and ordered TiO2 nanotube arrays for hydrogen generation. Int. J. Hydrog. Energy.

[7] Sivula K., Le Formal F., Grätzel M. (2011). Solar water splitting: Progress using hematite (α-Fe_2_O_3_) photoelectrodes. ChemSusChem.

[8] Cesar I., Kay A., Gonzalez Martinez J.A., Grätzel M. (2006). Translucent thin film Fe_2_O_3_ photoanodes for efficient water splitting by sunlight: Nanostructure-directing effect of Si-doping. J. Am. Chem. Soc..

[9] Liu Y., Xu Z., Yin M., Fan H., Cheng W., Lu L., Song Y., Ma J., Zhu X. (2015). Enhanced photoelectrocatalytic performance of α-Fe_2_O_3_ thin films by surface plasmon resonance of Au nanoparticles coupled with surface passivation by atom layer deposition of Al_2_O_3_. Nanoscale Res. Lett..

[10] Wang D., Li R., Zhu J., Shi J., Han J., Zong X., Li C. (2012). Photocatalytic water oxidation on BiVO_4_ with the electrocatalyst as an oxidation cocatalyst: Essential relations between electrocatalyst and photocatalyst. J. Phys. Chem. C.

[11] Jo W.J., Jang J.W., Kong K.-j., Kang H.J., Kim J.Y., Jun H., Parmar K., Lee J.S. (2012). Phosphate doping into monoclinic BiVO4 for enhanced photoelectrochemical water oxidation activity. Angew. Chem..

[12] Wang D., Jiang H., Zong X., Xu Q., Ma Y., Li G., Li C. (2011). Crystal facet dependence of water oxidation on BiVO_4_ sheets under visible light irradiation. Chem. A Eur. J..

[13] Steinfeld A. (2002). Solar hydrogen production via a two-step water-splitting thermochemical cycle based on Zn/ZnO redox reactions. Int. J. Hydrog. Energy.

[14] Wolcott A., Smith W.A., Kuykendall T.R., Zhao Y., Zhang J.Z. (2009). Photoelectrochemical study of nanostructured ZnO thin films for hydrogen generation from water splitting. Adv. Funct. Mater..

[15] Maeda K., Domen K. (2010). Solid solution of GaN and ZnO as a stable photocatalyst for overall water splitting under visible light. Chem. Mater..

[16] Tacca A., Meda L., Marra G., Savoini A., Caramori S., Cristino V., Bignozzi C.A., Pedro V.G., Boix P.P., Gimenez S. (2012). Photoanodes based on nanostructured WO_3_ for water splitting. ChemPhysChem.

[17] Hameed A., Gondal M., Yamani Z. (2004). Effect of transition metal doping on photocatalytic activity of WO_3_ for water splitting under laser illumination: Role of 3d-orbitals. Catal. Commun..

[18] Enesca A., Duta A., Schoonman J. (2007). Study of photoactivity of tungsten trioxide (WO_3_) for water splitting. Thin Solid Films.

[19] Pala R.A., Leenheer A.J., Lichterman M., Atwater H.A., Lewis N.S. (2014). Measurement of minority-carrier diffusion lengths using wedge-shaped semiconductor photoelectrodes. Energy Environ. Sci..

[20] Coridan R.H., Arpin K.A., Brunschwig B.S., Braun P.V., Lewis N.S. (2014). Photoelectrochemical behavior of hierarchically structured Si/WO_3_ core–shell tandem photoanodes. Nano Lett..

[21] Wang Y., Shi H., Cui K., Zhang L., Ge S., Jinghua Y. (2020). Reversible electron storage in tandem photoelectrochemical cell for light driven unassisted overall water splitting. Appl. Catal. B Environ..

[22] Li Y., Zhang W., Qiu B. (2020). Enhanced surface charge separation induced by Ag nanoparticles on WO_3_ photoanode for photoelectrochemical water splitting. Chem. Lett..

[23] Jun J., Ju S., Moon S., Son S., Huh D., Liu Y., Kim K., Lee H. (2020). The optimization of surface morphology of Au nanoparticles on WO_3_ nanoflakes for plasmonic photoanode. Nanotechnology.

[24] Huang J., Zhang Y., Ding Y. (2017). Rationally designed/constructed CoOx/WO_3_ anode for efficient photoelectrochemical water oxidation. Acs Catal..

[25] Wang S.L., Mak Y.L., Wang S., Chai J., Pan F., Foo M.L., Chen W., Wu K., Xu G.Q. (2016). Visible–Near-Infrared-Light-Driven Oxygen Evolution Reaction with Noble-Metal-Free WO_2_–WO_3_ Hybrid Nanorods. Langmuir.

[26] Baek J.H., Kim B.J., Han G.S., Hwang S.W., Kim D.R., Cho I.S., Jung H.S. (2017). BiVO_4_/WO_3_/SnO_2_ Double-Heterojunction photoanode with enhanced charge separation and visible-transparency for bias-free solar water-splitting with a perovskite solar cell. ACS Appl. Mater. Interfaces.

[27] Liu Y., Wygant B.R., Mabayoje O., Lin J., Kawashima K., Kim J.-H., Li W., Li J., Mullins C.B. (2018). Interface engineering and its effect on WO3-based photoanode and tandem cell. ACS Appl. Mater. Interfaces.

[28] Lee W.J., Shinde P.S., Go G.H., Ramasamy E. (2011). Ag grid induced photocurrent enhancement in WO_3_ photoanodes and their scale-up performance toward photoelectrochemical H_2_ generation. Int. J. Hydrogen Energy.

[29] Zhang J., Zhu G., Liu W., Xi Y., Golosov D., Zavadski S., Melnikov S. (2020). 3D core-shell WO_3_@ α-Fe_2_O_3_ photoanode modified by ultrathin FeOOH layer for enhanced photoelectrochemical performances. J. Alloys Compd..

[30] Zhang L.J., Li S., Liu B.K., Wang D.J., Xie T.F. (2014). Highly efficient CdS/WO_3_ photocatalysts: Z-scheme photocatalytic mechanism for their enhanced photocatalytic H_2_ evolution under visible light. ACS Catal..

[31] Lou Z., Zhu M., Yang X., Zhang Y., Whangbo M.-H., Li B., Huang B. (2018). Continual injection of photoinduced electrons stabilizing surface plasmon resonance of non-elemental-metal plasmonic photocatalyst CdS/WO_3_− x for efficient hydrogen generation. Appl. Catal. B Environ..

[32] Islam S.Z., Reed A., Wanninayake N., Kim D.Y., Rankin S.E. (2016). Remarkable enhancement of photocatalytic water oxidation in N_2_/Ar plasma treated, mesoporous TiO_2_ films. J. Phys. Chem. C.

[33] Islam S.Z., Rankin S.E. (2016). Hydrazine-based synergistic Ti (III)/N doping of surfactant-templated TiO_2_ thin films for enhanced visible light photocatalysis. Mater. Chem. Phys..

[34] Hou D., Hu X., Ho W., Hu P., Huang Y. (2015). Facile fabrication of porous Cr-doped SrTiO_3_ nanotubes by electrospinning and their enhanced visible-light-driven photocatalytic properties. J. Mater. Chem. A.

[35] Kudo A., Miseki Y. (2009). Heterogeneous photocatalyst materials for water splitting. Chem. Soc. Rev..

[36] Takanabe K. (2015). Solar water splitting using semiconductor photocatalyst powders. Solar Energy for Fuels.

[37] Takanabe K. (2017). Photocatalytic water splitting: Quantitative approaches toward photocatalyst by design. ACS Catal..

[38] Fujishima A., Honda K. (1972). Electrochemical photolysis of water at a semiconductor electrode. Nature.

[39] Youngblood W.J., Lee S.-H.A., Maeda K., Mallouk T.E. (2009). Visible light water splitting using dye-sensitized oxide semiconductors. Acc. Chem. Res..

[40] Maeda K., Eguchi M., Lee S.-H.A., Youngblood W.J., Hata H., Mallouk T.E. (2009). Photocatalytic hydrogen evolution from hexaniobate nanoscrolls and calcium niobate nanosheets sensitized by ruthenium (II) bipyridyl complexes. J. Phys Chem. C.

[41] Islam S.Z., Reed A., Kim D.Y., Rankin S.E. (2016). N_2_/Ar plasma induced doping of ordered mesoporous TiO_2_ thin films for visible light active photocatalysis. Microporous Mesoporous Mater..

[42] Jafari T., Moharreri E., Amin A.S., Miao R., Song W., Suib S.L. (2016). Photocatalytic water splitting—the untamed dream: A review of recent advances. Molecules.

[43] Wang Q., Hisatomi T., Jia Q., Tokudome H., Zhong M., Wang C., Pan Z., Takata T., Nakabayashi M., Shibata N. (2016). Scalable water splitting on particulate photocatalyst sheets with a solar-to-hydrogen energy conversion efficiency exceeding 1%. Nat. Mater..

[44] Iwase A., Ng Y.H., Ishiguro Y., Kudo A., Amal R. (2011). Reduced graphene oxide as a solid-state electron mediator in Z-scheme photocatalytic water splitting under visible light. J. Am. Chem. Soc..

[45] Sayama K., Mukasa K., Abe R., Abe Y., Arakawa H. (2002). A new photocatalytic water splitting system under visible light irradiation mimicking a Z-scheme mechanism in photosynthesis. J. Photochem. Photobiol. A Chem..

[46] Matsumoto Y., Unal U., Tanaka N., Kudo A., Kato H. (2004). Electrochemical approach to evaluate the mechanism of photocatalytic water splitting on oxide photocatalysts. J. Solid State Chem..

[47] Afroz K., Moniruddin M., Bakranov N., Kudaibergenov S., Nuraje N. (2018). A heterojunction strategy to improve the visible light sensitive water splitting performance of photocatalytic materials. J. Mater. Chem. A.

[48] Wang Z., Yang G., Tan C.K., Nguyen T.D., Tok A.I.Y. (2019). Amorphous TiO_2_ coated hierarchical WO_3_ Nanosheet/CdS Nanorod arrays for improved photoelectrochemical performance. Appl. Surface Sc..

[49] Hill J.C., Choi K.-S. (2012). Effect of Electrolytes on the Selectivity and Stability of n-type WO_3_ Photoelectrodes for Use in Solar Water Oxidation. J. Phys. Chem. C.

[50] Maeda K., Domen K. (2010). Photocatalytic water splitting: Recent progress and future challenges. J. Phys. Chem. Lett..

[51] Bard A.J. (1979). Photoelectrochemistry and heterogeneous photo-catalysis at semiconductors. J. Photochem..

[52] Kato H., Sasaki Y., Iwase A., Kudo A. (2007). Role of iron ion electron mediator on photocatalytic overall water splitting under visible light irradiation using Z-scheme systems. Bull. Chem. Soc. Jpn..

[53] Miseki Y., Fujiyoshi S., Gunji T., Sayama K. (2013). Photocatalytic water splitting under visible light utilizing I_3_−/I− and IO_3_−/I− redox mediators by Z-scheme system using surface treated PtO x/WO_3_ as O_2_ evolution photocatalyst. Catal. Sci. Technol..

[54] Yu W., Chen J., Shang T., Chen L., Gu L., Peng T. (2017). Direct Z-scheme g-C_3_N_4_/WO_3_ photocatalyst with atomically defined junction for H_2_ production. Appl. Catal. B Environ..

[55] He K., Xie J., Luo X., Wen J., Ma S., Li X., Fang Y., Zhang X. (2017). Enhanced visible light photocatalytic H_2_ production over Z-scheme g-C_3_N_4_ nansheets/WO_3_ nanorods nanocomposites loaded with Ni (OH)x cocatalysts. Chin. J. Catal..

[56] Chen Y., Wang L., Gao R., Zhang Y.-C., Pan L., Huang C., Liu K., Chang X.-Y., Zhang X., Zou J.-J. (2019). Polarization-Enhanced direct Z-scheme ZnO-WO_3_-x nanorod arrays for efficient piezoelectric-photoelectrochemical Water splitting. Appl. Catal. B Environ..

[57] Sayama K., Mukasa K., Abe R., Abe Y., Arakawa H. (2001). Stoichiometric water splitting into H_2_ and O_2_ using a mixture of two different photocatalysts and an IO_3_−/I− shuttle redox mediator under visible light irradiation. Chem. Commun..

[58] Wang F., Di Valentin C., Pacchioni G. (2012). Doping of WO_3_ for photocatalytic water splitting: Hints from density functional theory. J. Phys. Chem. C.

[59] Hwang D.W., Kim J., Park T.J., Lee J.S. (2002). Mg-doped WO_3_ as a novel photocatalyst for visible light-induced water splitting. Catal. Lett..

[60] Higashimoto S., Ushiroda Y., Azuma M. (2008). Electrochemically assisted photocatalysis of hybrid WO_3_/TiO_2_ films: Effect of the WO_3_ structures on charge separation behavior. Top. Catal..

[61] Ke D., Liu H., Peng T., Liu X., Dai K. (2008). Preparation and photocatalytic activity of WO3/TiO2 nanocomposite particles. Mater. Lett..

[62] Cui X.F., Wang Y.J., Jiang G.Y., Zhao Z., Xu C.M., Wei Y.C., Duan A.J., Liu J., Gao J.S. (2014). A photonic crystal-based CdS-Au-WO_3_ heterostructure for efficient visible-light photocatalytic hydrogen and oxygen evolution dagger. Rsc Adv..

[63] Chen B., Zhang Z., Baek M., Kim S., Kim W., Yong K. (2018). An antenna/spacer/reflector based Au/BiVO_4_/WO_3_/Au nanopatterned photoanode for plasmon-enhanced photoelectrochemical water splitting. Appl. Catal. B Environ..

[64] Chatchai P., Murakami Y., Kishioka S.-Y., Nosaka A.Y., Nosaka Y. (2009). Efficient photocatalytic activity of water oxidation over WO_3_/BiVO_4_ composite under visible light irradiation. Electrochim. Acta.

[65] Lee M.G., Kim D.H., Sohn W., Moon C.W., Park H., Lee S., Jang H.W. (2016). Conformally coated BiVO4 nanodots on porosity-controlled WO_3_ nanorods as highly efficient type II heterojunction photoanodes for water oxidation. Nano Energy.

[66] Pihosh Y., Turkevych I., Mawatari K., Asai T., Hisatomi T., Uemura J., Tosa M., Shimamura K., Kubota J., Domen K. (2014). Nanostructured WO_3_/BiVO_4_ photoanodes for efficient photoelectrochemical water splitting. Small.

[67] Müller A., Kondofersky I., Folger A., Fattakhova-Rohlfing D., Bein T., Scheu C. (2017). Dual absorber Fe_2_O_3_/WO_3_ host-guest architectures for improved charge generation and transfer in photoelectrochemical applications. Mater. Res. Express.

[68] Feng X., Chen Y., Qin Z., Wang M., Guo L. (2016). Facile fabrication of sandwich structured WO_3_ nanoplate arrays for efficient photoelectrochemical water splitting. ACS Appl. Mater. Interfaces.

[69] Kalanur S.S., Hwang Y.J., Chae S.Y., Joo O.S. (2013). Facile growth of aligned WO_3_ nanorods on FTO substrate for enhanced photoanodic water oxidation activity. J. Mater. Chem. A.

[70] Fan X., Gao B., Wang T., Huang X., Gong H., Xue H., Guo H., Song L., Xia W., He J. (2016). Layered double hydroxide modified WO_3_ nanorod arrays for enhanced photoelectrochemical water splitting. Appl. Catal. A General.

[71] Chae S.Y., Lee C.S., Jung H., Joo O.-S., Min B.K., Kim J.H., Hwang Y.J. (2017). Insight into charge separation in WO_3_/BiVO_4_ heterojunction for solar water splitting. ACS Appl. Mater. Interfaces.

[72] Su J., Zhang T., Wang L. (2017). Engineered WO_3_ nanorods for conformal growth of WO_3_/BiVO_4_ core–shell heterojunction towards efficient photoelectrochemical water oxidation. J. Mater. Sci. Mater. Electron..

[73] Yang J., Li W., Li J., Sun D., Chen Q. (2012). Hydrothermal synthesis and photoelectrochemical properties of vertically aligned tungsten trioxide (hydrate) plate-like arrays fabricated directly on FTO substrates. J. Mater. Chem..

[74] Amano F., Li D., Ohtani B. (2010). Fabrication and photoelectrochemical property of tungsten (VI) oxide films with a flake-wall structure. Chem. Commun..

[75] Zeng Q., Li J., Bai J., Li X., Xia L., Zhou B. (2017). Preparation of vertically aligned WO_3_ nanoplate array films based on peroxotungstate reduction reaction and their excellent photoelectrocatalytic performance. Appl. Catal. B Environ..

[76] Nayak A.K., Sohn Y., Pradhan D. (2017). Facile green synthesis of WO_3_ H_2_O nanoplates and WO_3_ nanowires with enhanced photoelectrochemical performance. Cryst. Growth Design.

[77] Park M., Seo J.H., Song H., Nam K.M. (2016). Enhanced visible light activity of single-crystalline WO_3_ microplates for photoelectrochemical water oxidation. J. Phys. Chem. C.

[78] Yang H.G., Sun C.H., Qiao S.Z., Zou J., Liu G., Smith S.C., Cheng H.M., Lu G.Q. (2008). Anatase TiO_2_ single crystals with a large percentage of reactive facets. Nature.

[79] Wang X., Liu G., Wang L., Pan J., Lu G.Q.M., Cheng H.-M. (2011). TiO_2_ films with oriented anatase {001} facets and their photoelectrochemical behavior as CdS nanoparticle sensitized photoanodes. J. Mater. Chem..

[80] Zou J.-P., Wu D.-D., Luo J., Xing Q.-J., Luo X.-B., Dong W.-H., Luo S.-L., Du H.-M., Suib S.L. (2016). A strategy for one-pot conversion of organic pollutants into useful hydrocarbons through coupling photodegradation of MB with photoreduction of CO_2_. Acs Catal..

[81] Zhang D., Wang S., Zhu J., Li H., Lu Y. (2012). WO_3_ nanocrystals with tunable percentage of (0 0 1)-facet exposure. Appl. Catal. B Environ..

[82] Wang S., Chen H., Gao G., Butburee T., Lyu M., Thaweesak S., Yun J.-H., Du A., Liu G., Wang L. (2016). Synergistic crystal facet engineering and structural control of WO_3_ films exhibiting unprecedented photoelectrochemical performance. Nano Energy.

[83] Zhang J., Zhang P., Wang T., Gong J. (2015). Monoclinic WO3 nanomultilayers with preferentially exposed (002) facets for photoelectrochemical water splitting. Nano Energy.

[84] Dong P., Hou G., Xi X., Shao R., Dong F. (2017). WO 3-based photocatalysts: Morphology control, activity enhancement and multifunctional applications. Environ. Sci. Nano.

[85] Zheng J.Y., Song G., Hong J., Van T.K., Pawar A.U., Kim D.Y., Kim C.W., Haider Z., Kang Y.S. (2014). Facile fabrication of WO_3_ nanoplates thin films with dominant crystal facet of (002) for water splitting. Cryst. Growth Des..

[86] Navarro J.R., Mayence A., Andrade J., Lerouge F., Chaput F., Oleynikov P., Bergstrom L., Parola S., Pawlicka A. (2014). WO_3_ nanorods created by self-assembly of highly crystalline nanowires under hydrothermal conditions. Langmuir.

[87] Su J., Feng X., Sloppy J.D., Guo L., Grimes C.A. (2011). Vertically aligned WO_3_ nanowire arrays grown directly on transparent conducting oxide coated glass: Synthesis and photoelectrochemical properties. Nano Lett..

[88] Rao P.M., Cho I.S., Zheng X. (2013). Flame synthesis of WO_3_ nanotubes and nanowires for efficient photoelectrochemical water-splitting. Proc. Combust. Inst..

[89] Koo W.-T., Choi S.-J., Kim N.-H., Jang J.-S., Kim I.-D. (2016). Catalyst-decorated hollow WO_3_ nanotubes using layer-by-layer self-assembly on polymeric nanofiber templates and their application in exhaled breath sensor. Sens. Actuators B Chem..

[90] Chen D., Hou X., Wen H., Wang Y., Wang H., Li X., Zhang R., Lu H., Xu H., Guan S. (2009). The enhanced alcohol-sensing response of ultrathin WO_3_ nanoplates. Nanotechnology.

[91] Enferadi-Kerenkan A., Ello A.S., Do T.-O. (2017). Synthesis, organo-functionalization, and catalytic properties of tungsten oxide nanoparticles as heterogeneous catalyst for oxidative cleavage of oleic acid as a model fatty acid into diacids. Ind. Eng. Chem. Res..

[92] Abdullin K.A., Kalkozova Z.K., Markhabayeva A.A., Dupre R., Moniruddin M., Nuraje N. (2018). Core–Shell (W@ WO_3_) Nanostructure to Improve Electrochemical Performance. ACS Appl. Energy Mater..

[93] Ma J., Zhang J., Wang S., Wang T., Lian J., Duan X., Zheng W. (2011). Topochemical preparation of WO_3_ nanoplates through precursor H_2_WO_4_ and their gas-sensing performances. J. Phys. Chem. C.

[94] Meng D., Wang G., San X., Song Y., Shen Y., Zhang Y., Wang K., Meng F. (2015). Synthesis of WO_3_ flower-like hierarchical architectures and their sensing properties. J. Alloys Compd..

[95] Adhikari S., Sarkar D. (2014). Hydrothermal synthesis and electrochromism of WO_3_ nanocuboids. RSC Adv..

[96] Ham D.J., Phuruangrat A., Thongtem S., Lee J.S. (2010). Hydrothermal synthesis of monoclinic WO_3_ nanoplates and nanorods used as an electrocatalyst for hydrogen evolution reactions from water. Chem. Eng. J..

[97] Jiao Z., Wang J., Ke L., Sun X.W., Demir H.V. (2011). Morphology-tailored synthesis of tungsten trioxide (hydrate) thin films and their photocatalytic properties. ACS Appl. Mater. Interfaces.

[98] Nagy D., Nagy D., Szilágyi I.M., Fan X. (2016). Effect of the morphology and phases of WO 3 nanocrystals on their photocatalytic efficiency. RSC Adv..

[99] Sieb N.R., Wu N.-c., Majidi E., Kukreja R., Branda N.R., Gates B.D. (2009). Hollow metal nanorods with tunable dimensions, porosity, and photonic properties. Acs Nano.

[100] Lu X., Wang G., Zhai T., Yu M., Gan J., Tong Y., Li Y. (2012). Hydrogenated TiO_2_ nanotube arrays for supercapacitors. Nano Lett..

[101] Kalanoor B.S., Seo H., Kalanur S.S. (2018). Recent developments in photoelectrochemical water-splitting using WO_3_/BiVO_4_ heterojunction photoanode: A review. Mater. Sci. Energy Technol..

[102] Pihosh Y., Turkevych I., Mawatari K., Uemura J., Kazoe Y., Kosar S., Makita K., Sugaya T., Matsui T., Fujita D. (2015). Photocatalytic generation of hydrogen by core-shell WO_3_/BiVO_4_ nanorods with ultimate water splitting efficiency. Sci. Rep..

[103] Hammad A., El-Bery H.M., El-Shazly A., Elkady M. (2018). Effect of WO_3_ morphological structure on its photoelectrochemical properties. Int. J. Electrochem. Sci.

[104] Zhou J., Lin S., Chen Y., Gaskov A. (2017). Facile morphology control of WO_3_ nanostructure arrays with enhanced photoelectrochemical performance. Appl. Surface Sci..

[105] Perera D., Lorek R., Khnayzer R.S., Moroz P., O’Connor T., Khon D., Diederich G., Kinder E., Lambright S., Castellano F.N. (2012). Photocatalytic activity of core/shell semiconductor nanocrystals featuring spatial separation of charges. J. Phys. Chem. C.

[106] Rao P.M., Cai L., Liu C., Cho I.S., Lee C.H., Weisse J.M., Yang P., Zheng X. (2014). Simultaneously efficient light absorption and charge separation in WO_3_/BiVO_4_ core/shell nanowire photoanode for photoelectrochemical water oxidation. Nano Lett..

[107] Jin B., Jung E., Ma M., Kim S., Zhang K., Kim J.I., Son Y., Park J.H. (2018). Solution-processed yolk–shell-shaped WO_3_/BiVO_4_ heterojunction photoelectrodes for efficient solar water splitting. J. Mater. Chem. A.

[108] Li Z., Zheng G., Wang J., Li H., Wu J., Du Y. (2016). Refining waste hardmetals into tungsten oxide nanosheets via facile method. J. Nanoparticle Res..

[109] Tan Y., Wang H., Liu P., Shen Y., Cheng C., Hirata A., Fujita T., Tang Z., Chen M. (2016). Versatile nanoporous bimetallic phosphides towards electrochemical water splitting. Energy Environ. Sci..

[110] Kim H., Hwang D., Kim Y., Lee J. (1999). Highly donor-doped (110) layered perovskite materials as novel photocatalysts for overall water splitting. Chem. Commun..

[111] Lee C.Y., Wang L., Kado Y., Killian M.S., Schmuki P. (2014). Anodic nanotubular/porous hematite photoanode for solar water splitting: Substantial effect of iron substrate purity. ChemSusChem.

[112] Troitskaia I., Gavrilova T., Atuchin V. (2012). Structure and micromorphology of titanium dioxide nanoporous microspheres formed in water solution. Phys. Procedia.

[113] Fan X., Liu Y., Chen S., Shi J., Wang J., Fan A., Zan W., Li S., Goddard W.A., Zhang X.-M. (2018). Defect-enriched iron fluoride-oxide nanoporous thin films bifunctional catalyst for water splitting. Nat. Commun..

[114] Shin S., Han H.S., Kim J.S., Park I.J., Lee M.H., Hong K.S., Cho I.S. (2015). A tree-like nanoporous WO_3_ photoanode with enhanced charge transport efficiency for photoelectrochemical water oxidation. J. Mater. Chem. A.

[115] Fujimoto I., Wang N., Saito R., Miseki Y., Gunji T., Sayama K. (2014). WO3/BiVO4 composite photoelectrode prepared by improved auto-combustion method for highly efficient water splitting. Int. J. Hydrogen Energy.

[116] Choi K.-S. (2010). Shape effect and shape control of polycrystalline semiconductor electrodes for use in photoelectrochemical cells. J. Phys. Chem. Lett..

[117] Song K., Gao F., Yang W., Wang E., Wang Z., Hou H. (2018). WO_3_ mesoporous nanobelts towards efficient photoelectrocatalysts for water splitting. ChemElectroChem.

[118] Markhabayeva A.A., Moniruddin M., Dupre R., Abdullin K.A., Nuraje N. (2020). Designing of WO_3_@ Co_3_O_4_ Heterostructure to Enhance Photoelectrochemical Performances. J. Phys. Chem. A.

[119] Kronawitter C.X., Vayssieres L., Shen S., Guo L., Wheeler D.A., Zhang J.Z., Antoun B.R., Mao S.S. (2011). A perspective on solar-driven water splitting with all-oxide hetero-nanostructures. Energy Environ. Sci..

[120] Moniruddin M., Afroz K., Shabdan Y., Bizri B., Nuraje N. (2017). Hierarchically 3D assembled strontium titanate nanomaterials for water splitting application. Appl. Surface Sci..

[121] Shabdan Y., Ronasi A., Coulibaly P., Moniruddin M., Nuraje N. (2017). Engineered core-shell nanofibers for electron transport study in dye-sensitized solar cells. AIP Adv..

[122] Abdullin K.A., Azatkaliev A., Gabdullin M., Kalkozova Z.K., Mukashev B., Serikkanov A. (2018). Preparation of Nanosized Tungsten and Tungsten Oxide Powders. Phys. Solid State.

[123] Lisitsyna L., Denisov G., Dauletbekova A., Karipbayev Z.T., Markhabaeva A., Vaganov V., Lisitsyn V.M., Akilbekov A. (2017). Luminescence of LiF crystals doped with uranium. Proc. J. Phys. Conf. Ser..

[124] Moniruddin M., Oppong E., Stewart D., McCleese C., Roy A., Warzywoda J., Nuraje N. (2019). Designing CdS-Based Ternary Heterostructures Consisting of Co-Metal and CoOx Cocatalysts for Photocatalytic H_2_ Evolution under Visible Light. Inorg. Chem..

[125] De Tacconi N.R., Chenthamarakshan C., Rajeshwar K., Pauporté T., Lincot D. (2003). Pulsed electrodeposition of WO_3_–TiO_2_ composite films. Electrochem. Commun..

[126] Smith W., Wolcott A., Fitzmorris R.C., Zhang J.Z., Zhao Y. (2011). Quasi-core-shell TiO_2_/WO_3_ and WO_3_/TiO_2_ nanorod arrays fabricated by glancing angle deposition for solar water splitting. J. Mater. Chem..

[127] Bae S.W., Ji S.M., Hong S.J., Jang J.W., Lee J.S. (2009). Photocatalytic overall water splitting with dual-bed system under visible light irradiation. Int. J. Hydrogen Energy.

[128] Ohno T., Tanigawa F., Fujihara K., Izumi S., Matsumura M. (1998). Photocatalytic oxidation of water on TiO_2_-coated WO_3_ particles by visible light using Iron (III) ions as electron acceptor. J. Photochem. Photobiol. A Chem..

[129] Yang M., He H., Zhang H., Zhong X., Dong F., Ke G., Chen Y., Du J., Zhou Y. (2018). Enhanced photoelectrochemical water oxidation on WO_3_ nanoflake films by coupling with amorphous TiO_2_. Electrochim. Acta.

[130] Momeni M.M., Ghayeb Y., Davarzadeh M. (2015). Single-step electrochemical anodization for synthesis of hierarchical WO_3_–TiO_2_ nanotube arrays on titanium foil as a good photoanode for water splitting with visible light. J. Electroanal. Chem..

[131] Lai C.W., Sreekantan S. (2013). Preparation of hybrid WO_3_–TiO_2_ nanotube photoelectrodes using anodization and wet impregnation: Improved water-splitting hydrogen generation performance. Int. J. Hydrogen Energy.

[132] Kwon Y.T., Song K.Y., Lee W.I., Choi G.J., Do Y.R. (2000). Photocatalytic behavior of WO_3_-loaded TiO_2_ in an oxidation reaction. J. Catal..

[133] Sajjad A.K.L., Shamaila S., Tian B., Chen F., Zhang J. (2010). Comparative studies of operational parameters of degradation of azo dyes in visible light by highly efficient WOx/TiO_2_ photocatalyst. J. Hazard. Mater..

[134] Hong S.J., Lee S., Jang J.S., Lee J.S. (2011). Heterojunction BiVO_4_/WO_3_ electrodes for enhanced photoactivity of water oxidation. Energy Environ. Sci..

[135] Xia L., Bai J., Li J., Zeng Q., Li X., Zhou B. (2016). A highly efficient BiVO_4_/WO_3_/W heterojunction photoanode for visible-light responsive dual photoelectrode photocatalytic fuel cell. Appl. Catal. B Environ..

[136] Santato C., Odziemkowski M., Ulmann M., Augustynski J. (2001). Crystallographically oriented mesoporous WO_3_ films: Synthesis, characterization, and applications. J. Am. Chem. Soc..

[137] Grigioni I., Stamplecoskie K.G., Selli E., Kamat P.V. (2015). Dynamics of photogenerated charge carriers in WO_3_/BiVO_4_ heterojunction photoanodes. J. Phys. Chem. C.

[138] Pattengale B., Ludwig J., Huang J. (2016). Atomic insight into the W-doping effect on carrier dynamics and photoelectrochemical properties of BiVO_4_ photoanodes. J. Phys. Chem. C.

[139] Mali M.G., Yoon H., Kim M.-w., Swihart M.T., Al-Deyab S.S., Yoon S.S. (2015). Electrosprayed heterojunction WO_3_/BiVO_4_ films with nanotextured pillar structure for enhanced photoelectrochemical water splitting. Appl. Phys. Lett..

[140] Cesar I., Sivula K., Kay A., Zboril R., Graätzel M. (2008). Influence of feature size, film thickness, and silicon doping on the performance of nanostructured hematite photoanodes for solar water splitting. J. Phys. Chem. C.

[141] Sadtler B., Demchenko D.O., Zheng H., Hughes S.M., Merkle M.G., Dahmen U., Wang L.-W., Alivisatos A.P. (2009). Selective facet reactivity during cation exchange in cadmium sulfide nanorods. J. Am. Chem. Soc..

[142] Li Y., Zhang L., Liu R., Cao Z., Sun X., Liu X., Luo J. (2016). WO_3_@ α-Fe_2_O_3_ Heterojunction arrays with improved photoelectrochemical behavior for neutral ph water splitting. ChemCatChem.

[143] Luo W., Yu T., Wang Y., Li Z., Ye J., Zou Z. (2007). Enhanced photocurrent–voltage characteristics of WO_3_/Fe_2_O_3_ nano-electrodes. J. Phys. D Appl. Phys..

[144] Li Y., Feng J., Li H., Wei X., Wang R., Zhou A. (2016). Photoelectrochemical splitting of natural seawater with α-Fe_2_O_3_/WO_3_ nanorod arrays. Int. J. Hydrogen Energy.

[145] Kumar P., Singh M., Reddy G.B. (2019). Core-Shell WO_3_-WS_2_ Nanostructured Thin Films via Plasma Assisted Sublimation and Sulfurization. ACS Appl. Nano Mater..

[146] Zhang J., Liu Z., Liu Z. (2016). Novel WO_3_/Sb_2_S_3_ heterojunction photocatalyst based on WO_3_ of different morphologies for enhanced efficiency in photoelectrochemical water splitting. ACS Appl. Mater. Interfaces.

[147] Wang Y., Tian W., Chen L., Cao F., Guo J., Li L. (2017). Three-dimensional WO_3_ nanoplate/Bi_2_S_3_ nanorod heterojunction as a highly efficient photoanode for improved photoelectrochemical water splitting. ACS Appl. Mater. Interfaces.

[148] Wang P., Huang B., Dai Y., Whangbo M.-H. (2012). Plasmonic photocatalysts: Harvesting visible light with noble metal nanoparticles. Phys. Chem. Chem. Phys..

[149] Hu D., Diao P., Xu D., Wu Q. (2016). Gold/WO_3_ nanocomposite photoanodes for plasmonic solar water splitting. Nano Res..

[150] Warren S.C., Thimsen E. (2012). Plasmonic solar water splitting. Energy Environ. Sci..

[151] Thimsen E., Le Formal F., Gratzel M., Warren S.C. (2010). Influence of plasmonic Au nanoparticles on the photoactivity of Fe_2_O_3_ electrodes for water splitting. Nano Lett..

[152] Singh T., Müller R., Singh J., Mathur S. (2015). Tailoring surface states in WO_3_ photoanodes for efficient photoelectrochemical water splitting. Appl. Surface Sci..

[153] Li Y.Z., Liu Z., Guo Z., Ruan M., Li X., Liu Y. (2019). Efficient WO_3_ photoanode modified by Pt layer and plasmonic Ag for enhanced charge separation and transfer to promote photoelectrochemical performances. ACS Sustain. Chem. Eng..

[154] Xu S., Fu D., Song K., Wang L., Yang Z., Yang W., Hou H. (2018). One-dimensional WO_3_/BiVO_4_ heterojunction photoanodes for efficient photoelectrochemical water splitting. Chem. Eng. J..

[155] Wu P.D., Liu Z.F., Ruan M.N., Guo Z.G., Zhao L. (2019). Cobalt-phosphate modified Fe-Zn0.2Cd0.8S/CuSbS_2_ heterojunction photoanode with multiple synergistic effect for enhancing photoelectrochemical water splitting. Appl. Surface Sci..

[156] Kim H.I., Kim J., Kim W., Choi W. (2011). Enhanced Photocatalytic and Photoelectrochemical Activity in the Ternary Hybrid of CdS/TiO_2_/WO_3_ through the Cascadal Electron Transfer. J. Phys. Chem. C.

[157] Zeng Q.Y., Bai J., Li J.H., Li L.S., Xia L.G., Zhou B.X., Sun Y.G. (2018). Highly-stable and efficient photocatalytic fuel cell based on an epitaxial TiO_2_/WO_3_/W nanothorn photoanode and enhanced radical reactions for simultaneous electricity production and wastewater treatment. Appl. Energy.

[158] Liu C.H., Qiu Y.Y., Zhang J., Liang Q., Mitsuzaki N., Chen Z.D. (2019). Construction of CdS quantum dots modified g-C_3_N_4_/ZnO heterostructured photoanode for efficient photoelectrochemical water splitting. J. Photochem. Photobiol. A Chem..

[159] Liew S.L., Zhang Z., Goh T.W.G., Subramanian G.S., Seng H.L.D., Hor T.S.A., Luo H.K., Chi D.Z. (2014). Yb-doped WO_3_ photocatalysts for water oxidation with visible light. Int. J. Hydrogen Energy.

[160] Subramanyam P., Vinodkumar T., Nepak D., Deepa M., Subrahmanyam C. (2019). Mo-doped BiVO_4_@reduced graphene oxide composite as an efficient photoanode for photoelectrochemical water splitting. Catal. Today.

[161] Zhang K., Shi X.J., Kim J.K., Park J.H. (2012). Photoelectrochemical cells with tungsten trioxide/Mo-doped BiVO_4_ bilayers. Phys. Chem. Chem. Phys..

[162] Tian L., Yang X.F., Cui X.K., Liu Q.Q., Tang H. (2019). Fabrication of dual direct Z-scheme g-C_3_N_4_/MoS_2_/Ag_3_PO_4_ photocatalyst and its oxygen evolution performance. Appl. Surface Sci..

[163] Kim J.Y., Youn D.H., Kang K., Lee J.S. (2016). Highly Conformal Deposition of an Ultrathin FeOOH Layer on a Hematite Nanostructure for Efficient Solar Water Splitting. Angew. Chem. Int. Ed..

[164] Lhermitte C.R., Verwer J.G., Bartlett B.M. (2016). Improving the stability and selectivity for the oxygen-evolution reaction on semiconducting WO_3_ photoelectrodes with a solid-state FeOOH catalyst. J. Mater. Chem. A.

[165] Bai S.L., Yang X.J., Liu C.Y., Xiang X., Luo R.X., He J., Chen A.F. (2018). An Integrating Photoanode of WO_3_/Fe_2_O_3_ Heterojunction Decorated with NiFe-LDH to Improve PEC Water Splitting Efficiency. Acs Sustain. Chem. Eng..

[166] Davi M., Ogutu G., Schrader F., Rokicinska A., Kustrowski P., Slabon A. (2017). Enhancing Photoelectrochemical Water Oxidation Efficiency of WO_3_/alpha-Fe_2_O_3_ Heterojunction Photoanodes by Surface Functionalization with CoPd Nanocrystals. Eur. J. Inorg. Chem..

[167] Sun J., Sun L., Yang X., Bai S., Luo R., Li D., Chen A. (2020). Photoanode of coupling semiconductor heterojunction and catalyst for solar PEC water splitting. Electrochim. Acta.

[168] Tahir M., Siraj M., Tahir B., Umer M., Alias H., Othman N. (2020). Au-NPs embedded Z–scheme WO_3_/TiO_2_ nanocomposite for plasmon-assisted photocatalytic glycerol-water reforming towards enhanced H2 evolution. Appl. Surface Sci..

[169] Zhu Z.F., Yan Y., Li J.Q. (2015). Preparation of flower-like BiOBr-WO_3_-Bi_2_WO_6_ ternary hybrid with enhanced visible-light photocatalytic activity. J. Alloys Compd..

[170] Hou Y., Zuo F., Dagg A.P., Liu J.K., Feng P.Y. (2014). Branched WO_3_ nanosheet array with layered C_3_N_4_ heterojunctions and CoOx Nanoparticles as a flexible photoanode for efficient photoelectrochemical water oxidation. Adv. Mater..

[171] Yan H.J., Tian C.G., Wang L., Wu A.P., Meng M.C., Zhao L., Fu H.G. (2015). Phosphorus-modified tungsten nitride/reduced graphene oxide as a high-performance, non-noble-metal electrocatalyst for the hydrogen evolution reaction. Angew. Chem. Int. Ed..

[172] Liu C.J., Yang Y.H., Li W.Z., Li J., Li Y.M., Shi Q.L., Chen Q.Y. (2015). Highly Efficient Photoelectrochemical Hydrogen Generation Using ZnxBi_2_S_3_+x Sensitized Platelike WO_3_ Photoelectrodes. Acs Appl. Mater. Interfaces.

[173] Hu J.S., Ren L.L., Guo Y.G., Liang H.P., Cao A.M., Wan L.J., Bai C.L. (2005). Mass production and high photocatalytic activity of ZnS nanoporous nanoparticles. Angew. Chem. Int. Ed..

[174] Zhang X.L., Wang X., Wang D.F., Ye J.H. (2019). Conformal BiVO_4_-Layer/WO_3_-Nanoplate-Array Heterojunction Photoanode Modified with Cobalt Phosphate Cocatalyst for Significantly Enhanced Photoelectrochemical Performances. Acs Appl. Mater. Interfaces.

[175] Wang C.H., Zhang X.T., Yuan B., Wang Y.X., Sun P.P., Wang D., Wei Y.A., Liu Y.C. (2014). Multi-heterojunction photocatalysts based on WO3 nanorods: Structural design and optimization for enhanced photocatalytic activity under visible light. Chem. Eng. J..

[176] Ran L., Yin L.W. (2019). Ternary Hierarchical Cu_7_S_4_/TiO_2_/CoCr-LDH Heterostructured Nanorod Arrays with Multiphase Reaction Interfaces for More Efficient Photoelectrochemical Water Splitting. Adv. Mater. Interfaces.

[177] Song S.Y., Zhang Y., Xing Y., Wang C., Feng J., Shi W.D., Zheng G.L., Zhang H.J. (2008). Rectangular AgIn(WO_4_)(2) nanotubes: A promising photoelectric material. Adv. Funct. Mater..

[178] Ma Z.Z., Hou H.L., Song K., Fang Z., Wang L., Gao F.M., Yang Z.B., Tang B., Yang W.Y. (2018). Ternary WO_3_/Porous-BiVO_4_/FeOOH Hierarchical Architectures: Towards Highly Efficient Photoelectrochemical Performance. Chemelectrochem.

[179] Shi X.J., Choi Y., Zhang K., Kwon J., Kim D.Y., Lee J.K., Oh S.H., Kim J.K., Park J.H. (2014). Efficient photoelectrochemical hydrogen production from bismuth vanadate-decorated tungsten trioxide helix nanostructures. Nat. Commun..

[180] Beermann N., Vayssieres L., Lindquist S.E., Hagfeldt A. (2000). Photoelectrochemical studies of oriented nanorod thin films of hematite. J. Electrochem. Soc..

[181] Zhang L., Huang Y., Dai C.H., Liang Q.M., Yang P., Yang H.H., Yan J.H. (2019). Constructing ZnO/ZnCr_2_O_4_@TiO_2_-NTA Nanocomposite for Photovoltaic Conversion and Photocatalytic Hydrogen Evolution. J. Electron. Mater..

[182] Cai J.J., Li S., Qin G.W. (2019). Interface engineering of Co_3_O_4_ loaded CaFe_2_O_4_/Fe_2_O_3_ heterojunction for photoelectrochemical water oxidation. Appl. Surface Sci..

[183] Yu C.L., Chen F.Y., Zeng D.B., Xie Y., Zhou W.Q., Liu Z., Wei L.F., Yang K., Li D.H. (2019). A facile phase transformation strategy for fabrication of novel Z-scheme ternary heterojunctions with efficient photocatalytic properties. Nanoscale.

[184] Lv J., Zhao Z.Y., Li Z.S., Ye J.H., Zou Z.G. (2009). Preparation and photocatalytic property of LiCr(WO_4_)(2). J. Alloys Compd..

[185] Coridan R.H., Shaner M., Wiggenhorn C., Brunschwig B.S., Lewis N.S. (2013). Electrical and Photoelectrochemical Properties of WO_3_/Si Tandem Photoelectrodes. J. Phys. Chem. C.

[186] Wang H.L., Turner J.A. (2010). Characterization of Hematite Thin Films for Photoelectrochemical Water Splitting in a Dual Photoelectrode Device. J. Electrochem. Soc..

[187] Wang H.L., Deutsch T., Turner J.A. (2008). Direct water splitting under visible light with nanostructured hematite and WO_3_ photoanodes and a GaInP2 photocathode. J. Electrochem. Soc..

[188] Fountaine K.T., Atwater H.A. (2014). Mesoscale modeling of photoelectrochemical devices: Light absorption and carrier collection in monolithic, tandem, Si vertical bar WO_3_ microwires. Opt. Express.

[189] Xing Z., Shen S.H., Wang M., Ren F., Liu Y., Zheng X.D., Liu Y.C., Xiao X.H., Wu W., Jiang C.Z. (2014). Efficient enhancement of solar-water-splitting by modified “Z-scheme” structural WO_3_-W-Si photoelectrodes. Appl. Phys. Lett..

[190] Sherman B.D., Sheridan M.V., Wee K.R., Marquard S.L., Wang D.G., Alibabaei L., Ashford D.L., Meyer T.J. (2016). A Dye-Sensitized Photoelectrochemical Tandem Cell for Light Driven Hydrogen Production from Water. J. Am. Chem. Soc..

[191] Shi X., Jeong H., Oh S.J., Ma M., Zhang K., Kwon J., Choi I.T., Choi I.Y., Kim H.K., Kim J.K. (2016). Unassisted photoelectrochemical water splitting exceeding 7% solar-to-hydrogen conversion efficiency using photon recycling. Nat. Commun..

[192] Akple M.S., Chimmikuttanda S.P. (2018). A ternary Z-scheme WO_3_-Pt-CdS composite for improved visible-light photocatalytic H-2 production activity. J. Nanoparticle Res..

[193] Bhat S.S.M., Lee S.A., Suh J.M., Hong S.-P., Jang H.W. (2018). Triple Planar Heterojunction of SnO_2_/WO_3_/BiVO_4_ with Enhanced Photoelectrochemical Performance under Front Illumination. Appl. Sci. Basel.

[194] Yourey J.E., Kurtz J.B., Bartlett B.M. (2012). Water Oxidation on a CuWO_4_-WO_3_ Composite Electrode in the Presence of Fe(CN)(6) (3-): Toward Solar Z-Scheme Water Splitting at Zero Bias. J. Phys. Chem. C.

[195] Prasad U., Prakash J., Gupta S.K., Zuniga J., Mao Y.B., Azeredo B., Kannan A.N.M. (2019). Enhanced Photoelectrochemical Water Splitting with Er- and W-Codoped Bismuth Vanadate with WO_3_ Heterojunction-Based Two-Dimensional Photoelectrode. Acs Appl. Mater. Interfaces.

[196] Pan Q., Zhang H., Yang Y., Cheng C. (2019). 3D Brochosomes-Like TiO_2_/WO_3_/BiVO_4_ Arrays as Photoanode for Photoelectrochemical Hydrogen Production. Small.

[197] Ma W., Wu X., Huang K., Wang M., Fu R., Chen H., Feng S. (2019). A Co(OH)(x) nanolayer integrated planar WO_3_/Fe_2_O_3_ photoanode for efficient photoelectrochemical water splitting. Sustain. Energy Fuels.

[198] Salimi R., Alvani A.A.S., Mei B.T., Naseri N., Du S.F., Mul G. (2019). Ag-Functionalized CuWO_4_/WO_3_ nanocomposites for solar water splitting. New J. Chem..

[199] Peng B., Xia M.Y., Li C., Yue C.S., Diao P. (2018). Network Structured CuWO_4_/BiVO_4_/Co-Pi Nanocomposite for Solar Water Splitting. Catalysts.

[200] Leonard K.C., Nam K.M., Lee H.C., Kang S.H., Park H.S., Bard A.J. (2013). ZnWO_4_/WO_3_ Composite for Improving Photoelectrochemical Water Oxidation. J. Phys. Chem. C.

[201] Scarongella M., Gadiyar C., Strach M., Rimoldi L., Loiudice A., Buonsanti R. (2018). Assembly of -Cu_2_V_2_O_7_/WO_3_ heterostructured nanocomposites and the impact of their composition on structure and photoelectrochemical properties. J. Mater. Chem. C.

[202] Li K.Z., Zhang C., Liu A.J., Chu D.M., Zhang C.Y., Yang P., Du Y.K., Huang J. (2018). Mesoporous tungsten oxide modified by nanolayered manganese-calcium oxide as robust photoanode for solar water splitting. J. Colloid Interface Sci..

[203] Li Y.T., Liu Z.F., Zhang J., Guo Z.G., Xin Y., Zhao L. (2019). 1D/0D WO_3_/CdS heterojunction photoanodes modified with dual co-catalysts for efficient photoelectrochemical water splitting. J. Alloy. Compd..

[204] Cui Y., Pan L., Chen Y., Afzal N., Ullah S., Liu D.Y., Wang L., Zhang X.W., Zou J.J. (2019). Defected ZnWO4-decorated WO_3_ nanorod arrays for efficient photoelectrochemical water splitting. Rsc. Adv..

[205] Ma Z.Z., Song K., Wang L., Gao F.M., Tang B., Hou H.L., Yang W.Y. (2019). WO_3_/BiVO_4_ Type-II Heterojunction Arrays Decorated with Oxygen-Deficient ZnO Passivation Layer: A Highly Efficient and Stable Photoanode. Acs Appl. Mater. Interfaces.

[206] Lv Y., Liu Y.K., Chen C.M., Wang T.H., Zhang M. (2018). Octopus tentacles-like WO_3_/C@CoO as high property and long life-time electrocatalyst for hydrogen evolution reaction. Electrochim. Acta.

[207] Yuan K.P., Cao Q., Li X.Y., Chen H.Y., Deng Y.H., Wang Y.Y., Luo W., Lu H.L., Zhang D.W. (2017). Synthesis of WO_3_@ZnWO_4_@ZnO-ZnO hierarchical nanocactus arrays for efficient photoelectrochemical water splitting. Nano Energy.

[208] Lin H.S., Lin L.Y. (2017). Improving Visible-light Responses and Electric Conductivities by Incorporating Sb_2_S_3_ and Reduced Graphene Oxide in a WO_3_ Nanoplate Array for Photoelectrochemical Water Oxidation. Electrochim. Acta.

[209] Jamali S., Moshaii A. (2017). Improving photo-stability and charge transport properties of Cu_2_O/CuO for photo-electrochemical water splitting using alternate layers of WO_3_ or CuWO_4_ produced by the same route. Appl. Surface Sci..

[210] Zhang H.F., Zhou W.W., Yang Y.P., Cheng C.W. (2017). 3D WO3/BiVO4/Cobalt Phosphate Composites Inverse Opal Photoanode for Efficient Photoelectrochemical Water Splitting. Small.

[211] Kalanur S.S., Yoo I.H., Park J., Seo H. (2017). Insights into the electronic bands of WO_3_/BiVO_4_/TiO_2_, revealing high solar water splitting efficiency. J. Mater. Chem. A.

[212] Gao H.Q., Zhang P., Hu J.H., Pan J.M., Fan J.J., Shao G.S. (2017). One-dimenSional Z-scheme TiO2/WO3/Pt heterostructures for enhanced hydrogen generation. Appl. Surface Sci..

[213] Peng X.N., He C., Liu Q.Y., Wang X.N., Wang H.B., Zhang Y.J., Ma Q.C., Zhang K., Han Y.B., Wang H. (2016). Strategic Surface Modification of TiO_2_ nanorods by WO_3_ and TiCl_4_ for the Enhancement in Oxygen Evolution Reaction. Electrochim. Acta.

